# The impact of air quality on public attention to depressive emotions: an empirical examination based on Chinese urban panel data

**DOI:** 10.3389/fpsyg.2026.1969885

**Published:** 2026-10-16

**Authors:** Chao Wang, Haitao Zhang

**Affiliations:** 1 Chongqing Mental Health Center, Chongqing, China; 2 School of Economics and Business Administration, Chongqing University of Education, Chongqing, China

**Keywords:** air quality, Baidu Index, depressive emotions, urban panel data, weather condition

## Abstract

Depressive emotions have emerged as a critical public health concern, with their etiology closely linked to the natural environment, particularly air quality. Grounded in environmental stress theory and social support theory, this study utilizes panel data from 277 prefecture-level cities in China spanning 2011–2023 to theoretically analyze and empirically examine the effects of air quality on public attention to depressive emotions and the underlying mechanisms. The findings reveal that: First, improvements in air quality significantly reduce public attention to depressive emotions, a conclusion that remains robust across multiple robustness tests. Second, the unemployment rate serves as a partial mediator between air quality and public attention to depressive emotions, while healthcare workforce allocation and public fiscal expenditure effectively buffer the adverse psychological impacts of air quality deterioration. Third, the influence of air quality on public attention to depressive emotions exhibits significant urban heterogeneity, being more pronounced in eastern and central regions, cities with net population outflows, and mature and declining resource-based cities. By examining environmental public goods and social support systems, this study provides novel empirical evidence for understanding the relationship between air quality and public mental health, offering valuable insights for promoting synergy between environmental policies and social security policies.

## 1 Introduction

In the context of rapid global urbanization and industrialization, maintaining good air quality has become a central challenge in public health. Substantial epidemiological evidence indicates that long-term exposure to atmospheric pollutants such as fine particulate matter (PM2.5) is significantly associated with the incidence and mortality of respiratory and cardiovascular diseases (Zhang et al., 2020), with its physiological health hazards having achieved broad consensus in the international academic community. However, as a fundamental environmental condition, the influence of air quality extends beyond traditional physical health domains, equally affecting public psychological states and closely correlating with common mental disorders such as depression and anxiety. Recent research suggests that atmospheric pollutants may impact mental health through two pathways: on one hand, pollutants directly damage brain structure and function through biological mechanisms including inducing neuroinflammation, triggering oxidative stress, and disrupting neuroendocrine regulation (Braithwaite et al., 2019); on the other hand, sustained exposure to adverse air environments restricts outdoor activities, reduces social interactions, and triggers health-related concerns, thereby exacerbating emotional distress and psychological burden (Beemer et al., 2021).

Two features of this setting motivate the study, and both concern magnitudes that are easy to understate. Public attention to depressive emotions is the first. Depression affects an estimated 280 million people worldwide and ranks among the leading causes of years lived with disability (GBD 2019 Mental Disorders Collaborators, 2022); in China the lifetime prevalence of depressive disorders is close to 7%, yet only a small minority of affected individuals ever reach specialist care (Huang et al., 2019). Most emotional distress therefore never enters a clinical record, and it is exactly this sub-threshold pool that surfaces first as information seeking. Attention measured at population level accordingly does work that diagnosis rates cannot: it responds ahead of formal presentation, it registers help-seeking intention alongside symptoms, and it is observable continuously for every city rather than only for surveyed samples. Establishing what moves it has direct value for prevention planning and for the timing and targeting of mental health service supply (Figure 1).

**Figure 1:**
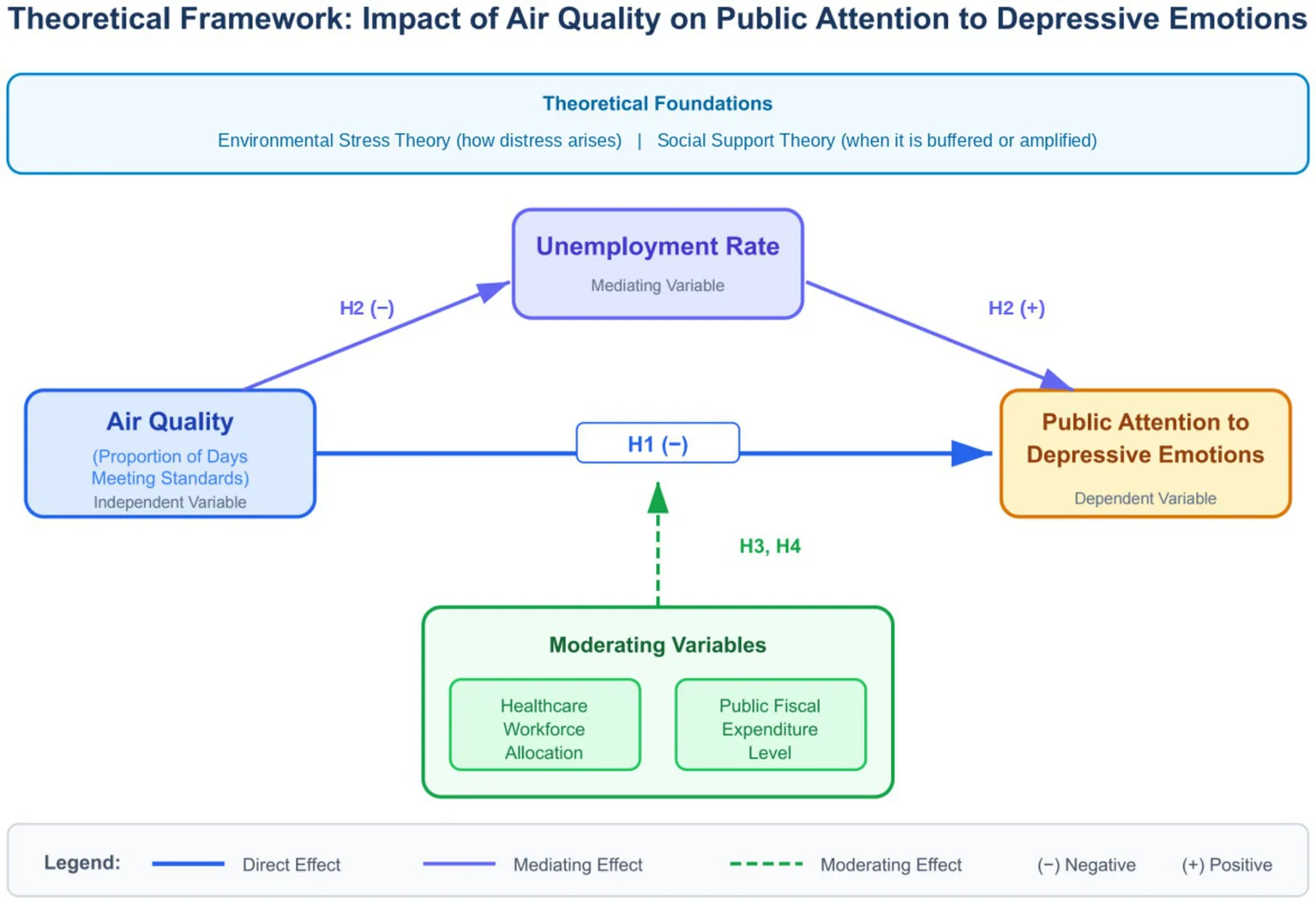
Theoretical framework diagram.

Air quality is the second. It has changed sharply and unevenly in China over the study window, which is what makes it informative rather than merely important. Following the Air Pollution Prevention and Control Action Plan of 2013 and the Blue Sky Protection Campaign of 2018, the mean share of days meeting the Grade II standard across the 120 cities with continuous monitoring records since 2011 (Figure 2) rose from roughly 67% in 2014 to roughly 82% in 2023, while the mean annual AQI fell from about 95 to about 75. Convergence has not accompanied this improvement. Central-region cities still average close to 78% compliant days, some seven to nine percentage points below the northeastern and western averages, and heavy pollution episodes recur each winter across the northern plain. A strong secular improvement combined with persistent cross-city disparity is precisely the configuration that allows air quality to be separated from national trends in a panel design.

**Figure 2:**
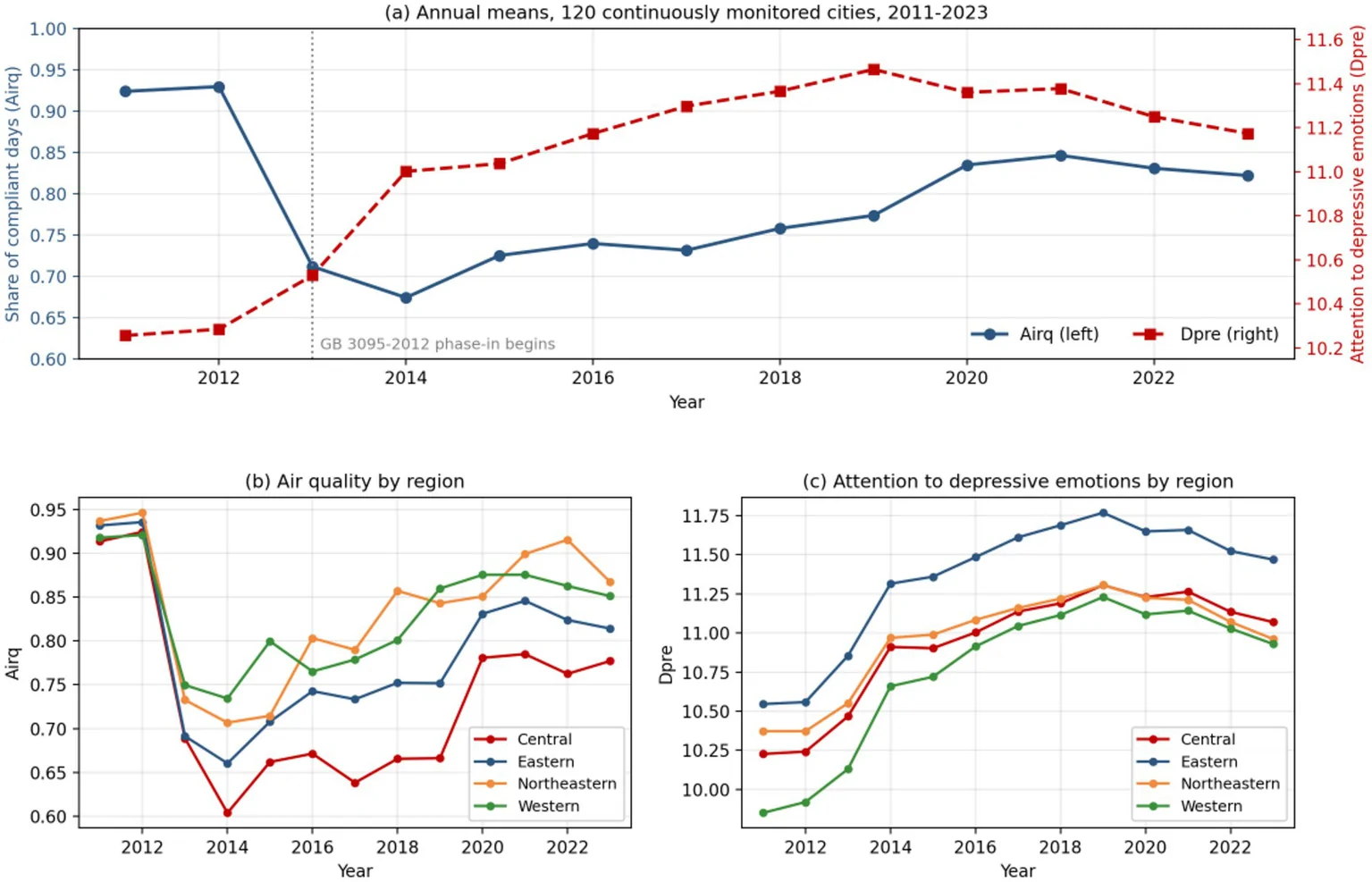
Temporal and Regional patterns of air quality and public attention to depressive emotions. All three panels are plotted on the 120 cities that have air quality monitoring records in every year from 2011 to 2023 (47 eastern, 26 central, 35 western and 12 northeastern). The other sample cities lack continuous monitoring records before 2014 or 2015, and their early-year values in the estimation sample come from the yearbook compliance counts described in Section 4.2, so this balanced subsample keeps the trends from being driven by changes in the set of cities covered as the monitoring network expanded. Because these cities have lower compliance shares and higher search intensity than the sample as a whole, their pooled means (Airq 0.793, Dpre 11.044) differ from the estimation-sample means in Table 7. Airq is the share of days meeting the Grade II standard and Dpre is the log of the summed Baidu search index for the three keywords. The vertical line in panel (a) marks 2013, the first year of AQI reporting under GB 3095–2012, which began in 74 key cities, covered 161 cities from 2014 and all prefecture-level and above cities from 2015; compliance is measured on the earlier API basis for all cities before 2013 and, in 2013 and 2014, for cities not yet reporting under the new standard.

Although existing literature has preliminarily explored the association between air quality and public attention to depressive emotions (Bhui et al., 2023), several methodological limitations persist. First, regarding data timeliness and measurement perspectives, traditional analyses have predominantly relied on pollutant concentration indicators or low-frequency health survey data, failing to reveal the sustained impact of clean air availability on public attention to depressive emotions from the dimension of air quality compliance days. Second, concerning measurement instruments and sample coverage, studies based on clinical diagnoses or self-report scales are often constrained by high costs and limited sample sizes, susceptible to reporting bias and social desirability effects, making continuous and objective monitoring of psychological states across large populations challenging.

The digital era provides new avenues for overcoming these bottlenecks. Internet search engines have become important channels for the public to express health concerns and explore symptom information (Barros et al., 2020). Individual internal psychological states, particularly emotional distress not yet reaching clinical recognition thresholds, are often sensitively reflected in specific online search behaviors. Baidu, as China’s search engine with the highest market share, generates the “Baidu Index” that captures massive users’ collective attention to specific keywords in real-time and anonymously, providing high-frequency, objective “digital footprints” for measuring group psychological states. Existing research has demonstrated that such search data can effectively track dynamic changes in public health events, economic uncertainty, and seasonal affective disorders (Liang et al., 2025), establishing the methodological foundation for using search volumes of “depression” and related terms as proxy indicators for public attention to depressive emotions.

Against this background, this study focuses on the following core questions: At the Chinese city level, does air quality—represented by the “proportion of days meeting air quality standards”—systematically correlate with public attention to depressive emotions? Through what mechanisms does this influence transmit? Does this influence exhibit heterogeneity across different types of cities? To address these questions, this study employs environmental stress theory and social support theory as analytical frameworks, utilizing panel data from 277 prefecture-level cities in China from 2011 to 2023 to theoretically analyze and empirically examine the impact of air quality on public attention to depressive emotions and its mechanisms. The main findings are as follows: First, air quality improvement significantly reduces public attention to depressive emotions, an effect that remains robust after controlling for multiple urban characteristics and meteorological conditions. Second, air quality indirectly affects public mental health through urban unemployment rates, while adequate healthcare workforce allocation and public fiscal expenditure effectively buffer the negative psychological impacts of poor air quality. Third, the influence of air quality on public attention to depressive emotions exhibits significant urban heterogeneity, being more pronounced in eastern and central regions, cities with net population outflows, and resource-dependent cities (particularly mature and declining types).

This study’s marginal contributions are reflected in three aspects. First, methodological innovation: applying the Baidu Index at the Chinese city level to construct a “public attention to depressive emotions” indicator, achieving dynamic and objective measurement of group psychological states, and providing new data sources and measurement paradigms for environmental mental health research. Second, theoretical extension: constructing an “environment-society-psychology” integrated analytical framework, incorporating macro factors such as unemployment rates, public healthcare services, and fiscal capacity into the model, and analyzing their mediating and moderating roles in the relationship between air quality and public attention to depressive emotions, thereby clarifying the pathways through which macro environmental risks transmit to individual mental health. Third, heterogeneity deepening: systematically revealing the differentiated manifestations of air quality’s impact on public attention to depressive emotions across regional development stages, population mobility structures, and urban resource types, identifying high-vulnerability areas for environmental psychological risk, and providing empirical evidence for precise and differentiated environmental health governance.

## 2 Literature review

Air quality is a comprehensive indicator measuring atmospheric environmental conditions, typically evaluated based on concentration levels of major pollutants including PM2.5, PM10, sulfur dioxide, and nitrogen dioxide (World Health Organization, 2021). Air pollution, as a negative representation of air quality, reflects the degree to which the atmospheric environment deviates from healthy benchmarks and constitutes a core dimension of environmental risk assessment. This logical chain from environmental exposure to health effects makes the mechanisms through which air quality and its pollution status affect public mental health a key topic for interdisciplinary research spanning environmental science, epidemiology, and psychology.

Extensive observational studies provide an empirical foundation for the association between air pollution and depressive emotions. Early research, primarily based on cross-sectional surveys of specific regions or populations, revealed positive associations between air pollutant exposure and depressive symptom scores or prevalence rates (Khosrorad et al., 2022). With the accumulation of longitudinal cohort studies, the relationship between long-term exposure to fine particulate matter (PM2.5) and increased depression incidence risk has been further confirmed (Braithwaite et al., 2019). A meta-analysis by Zeng et al. (2019) integrating multiple observational studies demonstrated that long-term PM2.5 exposure and short-term exposure to PM10, nitrogen dioxide (NO2), sulfur dioxide (SO2), and carbon monoxide (CO) are significantly associated with elevated depression risk. However, heterogeneity exists in the effects of different pollutants; for example, the impact of ozone (O3) on depression remains inconsistent across studies, suggesting that pollutant type, exposure time windows, and study design may moderate the ultimate effects (Zhang et al., 2026).

In-depth exploration of potential mechanisms is crucial for understanding these associations, with current research proceeding along two main pathways. First, the neurobiological pathway: Research indicates that air pollutants can impair blood–brain barrier integrity through inducing systemic inflammation and oxidative stress, activate microglia and trigger neuroinflammation, thereby damaging brain regions closely associated with emotional regulation (such as the hippocampus and prefrontal cortex), ultimately increasing depression risk (Block and Calderón-Garcidueñas, 2009). Recent studies have further validated this pathway through biomarkers: Zhang et al. (2026) found that the association between PM2.5 exposure and depressive symptoms is partially mediated by neuroinflammation markers (such as cerebrospinal fluid sTREM2), potentially involving Alzheimer’s disease-related pathological processes. Second, the socio-psychological and behavioral pathway: This pathway focuses on how air pollution indirectly damages mental health by affecting cognition, behavior, and social interaction (Ventriglio et al., 2021). Theory suggests that sustained air pollution restricts residents’ outdoor activities, reduces social interactions, triggers persistent health concerns, and causes sensory discomfort (such as reduced visibility), factors that may induce helplessness, intensify psychological stress, and diminish perceived quality of life, thereby precipitating or exacerbating depressive emotions (Hahad et al., 2020).

Existing research consistently finds that the mental health effects of air pollution exhibit significant heterogeneity (Cao et al., 2023), primarily moderated by individual characteristics and macro-contextual factors. At the individual level, genetic makeup is a key moderating factor. Gene–environment interaction studies have found that individuals carrying specific high-risk genotypes show significantly amplified depression risk under pollution exposure (Sun et al., 2025). Additionally, life cycle stage (such as children/adolescents, middle-aged/elderly), gender (females typically at higher risk), and pre-existing health conditions are important vulnerability factors (Qiu et al., 2023). At the macro level, socioeconomic status and community environmental resources play buffering or exacerbating roles. Research indicates that groups facing environmental injustice, such as low-income populations and minority communities, often bear higher pollution exposure and mental health risks (Burrows et al., 2025); while high-quality community resources (such as adequate green spaces) can effectively mitigate the negative impacts of air pollution on mental health, with protective effects particularly pronounced for genetically susceptible individuals (Sun et al., 2025).

The evolution of research methods has also profoundly influenced this field’s development. Traditionally, depression outcome measurement has primarily relied on clinical diagnostic records, structured psychological scales, or prescription data (Fried et al., 2022). While these methods possess high validity, they also have obvious limitations: medical data coverage is incomplete with inherent lag, scale surveys are costly and difficult to implement frequently, all constraining the ability to dynamically and real-time monitor psychological states across large populations. To overcome these bottlenecks, research frontiers are actively introducing innovative data and methods. In terms of causal identification, instrumental variable methods and quasi-experimental designs (such as leveraging policy shocks or meteorological condition changes) are widely employed to enhance the robustness of causal inference (Zhao et al., 2025). Regarding measurement instruments, using digital footprint data (such as social media sentiment and online search behavior) as real-time proxy indicators for group psychological states has become a promising research direction. Such data possess advantages of high frequency, objectivity, and broad coverage, effectively capturing public collective attention to health issues and emotional fluctuations, providing new possibilities for exploring the dynamic relationship between environment and psychology at large spatiotemporal scales.

In summary, existing literature has established the status of air pollution as a risk factor for depressive emotions and made important advances in biological mechanisms and individual heterogeneity. However, the following research gaps remain to be addressed: First, at the measurement level, research using real-time behavioral big data (such as search engine indices) for high-frequency, macro-level monitoring of public attention to depressive emotions remains nascent, particularly requiring strengthened application and validation in the Chinese context. Second, at the mechanism level, existing research predominantly focuses on micro-biological pathways, with relatively insufficient empirical studies systematically examining mechanisms from macro urban socioeconomic structural perspectives (such as unemployment rates, public finance, and healthcare service allocation). Third, at the heterogeneity analysis level, existing research emphasizes individual-level differences, lacking systematic examination of city-level structural heterogeneity (such as regional development stages, population mobility patterns, and resource dependence).

## 3 Theoretical analysis and research hypotheses

### 3.1 Direct impact of air quality on public attention to depressive emotions

The direct effect is developed from two theories rather than a catalogue of perspectives. Environmental stress theory specifies how a persistent environmental condition becomes a psychological load; social support theory specifies why that load is not absorbed when the resource lost is one that everyone shares. The two are complementary rather than parallel. The first identifies the mechanism that generates distress, the second the conditions under which distress is buffered or amplified. Taken together they deliver a single prediction for the direct effect and a stated rationale for the moderators tested later, and neither is invoked merely to add a label.

From the environmental stress theory perspective, air pollution constitutes a typical chronic, diffuse environmental stressor. According to classical stress theory (Folkman et al., 1986), stress arises from individuals’ cognitive appraisal of imbalance between environmental demands and their coping resources. The physical characteristics of air pollution (such as elevated PM2.5 concentrations) and sensory signals (such as reduced visibility and pervasive odors), as persistent environmental stimuli, are first perceived by the public and initiate the cognitive appraisal process. In the primary appraisal stage, individuals judge polluted weather as threatening to their health, safety, and autonomous activities; in the secondary appraisal stage, due to the regional and persistent nature of pollution, individuals generally develop a “sense of coping powerlessness,” perceiving themselves as unable to effectively change or escape this environment. This sustained threatening appraisal and low sense of control cognition constitute the core psychological mechanisms inducing negative emotions such as anxiety, helplessness, and depression (Arnsten, 2015). From a neurobiological perspective, chronic environmental stress leads to hypothalamic–pituitary–adrenal (HPA) axis dysregulation, suppresses prefrontal cortex function (responsible for higher cognition and emotional regulation), while over-activating the amygdala (responsible for fear and anxiety responses)—these neuroendocrine changes collectively form the physiological basis for depressive emotion generation. Furthermore, air pollutants can directly penetrate the blood–brain barrier, damaging emotion-regulating brain regions such as the hippocampus and prefrontal cortex through pathways including inducing neuroinflammation and oxidative stress, further exacerbating depression risk. Therefore, air pollution is not merely a physical environmental hazard but an important risk factor directly damaging mental health through sustained activation of stress responses (Thomson, 2019).

Three features distinguish air pollution from the acute stressors on which most of this literature rests, and they explain why its psychological cost accumulates. It is chronic rather than episodic, so no recovery interval follows exposure. It is uncontrollable at the individual level, so problem-focused coping is largely unavailable and residents fall back on emotion-focused coping, which is the strategy most consistently linked to depressive rather than anxious outcomes. And it is ambient, so avoidance requires withdrawal from ordinary daily activity rather than avoidance of a discrete event. The result is a low-grade but continuous demand on coping resources, and that is the configuration under which appraisal models predict depressed mood rather than acute alarm.

From the social support theory perspective, the absence of clean air is better understood as the loss of a support resource than as one more stressor (Ntontis et al., 2020). The theory distinguishes a direct pathway, in which resources raise well-being irrespective of stress exposure, from a buffering pathway, in which resources weaken the link between a stressor and its psychological consequences (Thoits, 1995). Clean air operates on both. It is the most widely shared environmental resource a city supplies, requiring no solicitation, no eligibility and no individual outlay, and it underwrites the physiological comfort and perceived safety on which ordinary positive affect rests. Chronic scarcity weakens the direct pathway because a baseline welfare input is withdrawn from every resident at once.

The buffering pathway is weakened simultaneously, and this is the less obvious loss. Support is produced largely through interaction, and interaction is what polluted air suppresses. Outdoor recreation, exercise, informal neighborhood contact and the routine co-presence from which instrumental and emotional support are drawn all decline when residents stay indoors, so the resource that would otherwise absorb environmental stress contracts exactly when demand for it rises (Hazlehurst et al., 2018). Because the exposure is regional, the contraction is collective: neighbors, family members and colleagues are constrained at the same time, and support cannot be redistributed from unaffected members of the same network in the way it can after an individual-level shock. Environmental deprivation and support atrophy thus reinforce one another, and that reciprocal process is how a physical environmental condition is converted into a durable deterioration in collective emotional state.

Based on the above analysis, we propose the core research hypothesis H1:

*H1*: Air quality is significantly negatively correlated with public attention to depressive emotions; higher air quality corresponds to lower levels of public attention to depressive emotions.

### 3.2 Mediating effect of unemployment rate

The impact of air quality on public mental health may be indirectly transmitted through urban labor market conditions, with unemployment rate constituting an important mediating variable.

First, regarding the mechanisms through which air quality affects urban unemployment rates (Li et al., 2020): From the labor supply side, long-term exposure to air-polluted environments damages workers’ respiratory and cardiovascular health, leading to increased absenteeism due to illness and reduced work capacity, with some workers potentially forced to exit the labor market due to health reasons (Hanna and Oliva, 2015). From the labor demand side, air quality is an important component of urban livability and business environment; persistent heavy pollution weather reduces cities’ attractiveness to high-quality talent and knowledge-intensive enterprises, constraining service sector and emerging industry development, thereby affecting job creation capacity (Khanna et al., 2025). The two sides reinforce one another, so a sustained deterioration in air quality is expected to raise the local unemployment rate even though the supply-side and demand-side responses differ in timing.

Three points about this first link deserve to be explicit. The channels differ in speed. Health-driven reductions in labor supply operate within the year through absenteeism and reduced effective hours, whereas firms’ location and investment decisions respond with a lag of one or more years, which is consistent with the persistence of the lagged estimates reported in Table 1. The channels also differ in incidence. Pollution-related health losses fall disproportionately on outdoor, manual and informal workers, who hold the least contractual protection and face the weakest re-employment prospects, so a given rise in the city unemployment rate carries more psychological weight than the headline figure suggests. And the two sides interact. As polluted cities lose their most mobile high-skilled workers, the local skill composition thins, which further deters entry by knowledge-intensive employers and turns an environmental disadvantage into a self-reinforcing labor-market disadvantage.

**Table 1:** Robustness tests considering lagged effects of core variables.

Variables	**(1)**	**(2)**	**(3)**
	Dpre	Dpre	Dpre
L1.Airq	−0.816***		
	(−12.425)		
L2.Airq		−0.544***	
		(−8.503)	
L3.Airq			−0.523***
			(−3.954)
RGDP	0.000***	0.000*	0.000
	(6.904)	(1.940)	(1.043)
Nid	−0.000	0.000	0.000
	(−0.425)	(0.363)	(0.463)
Nfdi	0.001***	0.001***	0.000*
	(3.397)	(2.625)	(1.885)
Tue	0.000***	0.000***	0.000***
	(3.440)	(3.943)	(3.791)
Shoh	−0.001***	−0.001***	−0.001***
	(−5.679)	(−4.799)	(−4.382)
Mpu	0.000***	0.000***	0.000***
	(5.025)	(3.565)	(3.013)
Npe	0.000	0.000	0.000
	(0.395)	(0.891)	(1.147)
Tsoe	−0.000***	−0.000***	−0.000***
	(−16.644)	(−13.616)	(−8.417)
City FE	YES	YES	YES
Year FE	YES	YES	YES
Constant	10.896***	10.932***	10.798***
	(125.431)	(128.004)	(138.529)
Observations	3324	3047	2770
*R*-squared	0.487	0.419	0.324
Number of id	277	277	277

*t*-statistics in parentheses. ***p < 0.01, **p < 0.05, *p < 0.1.

Second, regarding the mechanisms through which unemployment rates affect public attention to depressive emotions: The damage of unemployment to individual mental health has been confirmed in numerous studies (Paul and Moser, 2009). Unemployment signifies not only loss of economic income and declining material living standards but also accompanies psychological impacts including loss of social roles, collapse of daily life structures, and shaken self-worth. Jahoda's (1982) latent deprivation theory points out that employment, beyond providing income, also satisfies individuals’ psychological needs for time structure, social interaction, collective goals, identity, and regular activity—unemployment means simultaneous loss of these “latent functions.” At the city level, higher unemployment rates reflect regional economic stagnation and accumulated social pressure; even employed individuals may experience anxiety due to concerns about unemployment risk.

Two refinements matter for the mechanism examined here. The psychological cost of unemployment is not confined to the unemployed: job insecurity among those still working produces distress of comparable magnitude to unemployment itself, and a rising city unemployment rate is the most visible signal of that insecurity, so a city-level rate captures an exposure far broader than the headcount out of work. The cost is also transmitted within households, since the loss of a single earner alters the material circumstances and the emotional climate of every member, including those outside the labor force altogether. Both refinements imply that the unemployment channel should be detectable in aggregate search behavior rather than only in the records of affected individuals, which is what the mediation test reported below examines.

Based on the above analysis, we propose research hypothesis H2:

*H2*: Urban unemployment rate mediates the relationship between the proportion of days meeting air quality standards and public attention to depressive emotions.

### 3.3 Moderating effect of healthcare workforce

Healthcare workforce size, as a core indicator of public health service capacity, may play an important moderating role in the relationship between air quality and public attention to depressive emotions. The theoretical basis can be elaborated from the following two dimensions.

First, health assurance and risk buffering mechanisms: Adequate healthcare personnel signifies higher healthcare service accessibility and stronger health problem response capabilities (Aljuaid et al., 2022). When residents live in air-polluted environments, concerns about health risks such as respiratory diseases represent an important pathway triggering anxiety and depression. Sufficient healthcare personnel resources can significantly reduce the strength of the “pollution—health risk—psychological stress” transmission chain through providing timely health consultation, diagnostic services, and preventive care. When residents perceive stronger institutional protection for their health, their vulnerability and helplessness regarding environmental hazards correspondingly diminish, thereby weakening the direct impact of air pollution on mental health.

Second, resource complementarity and synergistic enhancement mechanisms: Clean air and quality healthcare services can be viewed as two types of key input factors in producing the final product of “health” (Eckelman et al., 2018). According to health production function theory, complementary effects may exist between different factors. Good air quality can reduce the incidence of underlying diseases, while abundant healthcare personnel resources ensure efficient disease treatment and promote broader health promotion efforts. The synergistic effect of both can more substantially enhance residents’ overall health levels and sense of life security, with the combined psychological well-being benefits potentially exceeding the sum of their independent effects (Phillips et al., 2005). In other words, in cities with higher healthcare service levels, the marginal mental health benefits from air quality improvement may be greater, as comprehensive healthcare systems can more fully translate the “health dividends” of environmental improvement into residents’ perceived security and well-being.

Based on the above analysis, we propose hypothesis H3:

*H3*: Urban healthcare workforce size positively moderates the impact of air quality on public attention to depressive emotions.

### 3.4 Moderating effect of urban public fiscal expenditure

Public fiscal expenditure, as the core instrument for urban governments to allocate resources and fulfill public functions, is a key macro factor shaping urban socioeconomic development environments and residents’ quality of life (Balynskaya and Vasilyeva, 2017). Higher fiscal expenditure levels, particularly spending directed toward public services and livelihood areas, indicate governments possess stronger capabilities and resources to address various social challenges including environmental issues, thereby potentially buffering the negative impacts of external environmental pressures on residents’ mental health.

According to Hobfoll's (1989) Conservation of Resources Theory, the more abundant the resources possessed by individuals or communities, the stronger their capacity to resist and adapt to stressors. At the city level, abundant public fiscal resources constitute precisely such a key macro “protective resource,” providing material foundations for implementing effective interventions and constructing social safety nets. Specifically, urban public fiscal expenditure may moderate the impact of air quality on public attention to depressive emotions through the following pathways.

First, environmental governance and infrastructure improvement: Stronger fiscal expenditure capacity can directly translate into more effective environmental governance investment (Wu et al., 2020), such as optimizing public transportation systems, promoting clean energy, and expanding urban green spaces—measures that can fundamentally improve air quality or mitigate its adverse effects. Second, public service provision and psychological compensation: High-quality public services supported by fiscal resources, including comprehensive social security systems, accessible public cultural facilities, and broadly covered community service networks, can provide residents with alternative psychological support and leisure options, thereby partially compensating for psychological losses caused by restricted outdoor activities due to poor air quality. Third, macroeconomic stability and stress relief: Strong fiscal capacity helps maintain good macroeconomic environments and social stability (Ocampo, 2008), relieving psychological pressure exacerbated by economic uncertainty or unemployment risk through promoting employment, stabilizing prices, and providing social assistance, thereby weakening the association between environmental problems and psychological distress.

Based on the above analysis, we propose hypothesis H4:

*H4*: Urban public fiscal expenditure levels significantly moderate the impact of the proportion of days meeting air quality standards on public attention to depressive emotions.

## 4 Model construction and variable selection

### 4.1 Model construction

To test the relationship between air quality and public attention to depressive emotions, the model is specified as follows:

Dpreit=α+βAirqit+ρXit+μi+λt+εit (1)

where i denotes cities, t denotes years; Dpre represents public attention to depressive emotions; Airq represents air quality; X represents control variables; μ represents city fixed effects, λ represents time fixed effects; ε represents the random error term; α, β, and ρ represent estimated coefficients.

Equation 1 is estimated by two-way fixed-effects least squares on a balanced panel in which the unit of observation is the city-year: 277 prefecture-level cities observed in each of the 13 years from 2011 to 2023, giving 3,601 observations. No further pooling or averaging is applied. The only aggregation in constructing the data is that of the daily AQI and Baidu Index series to the city-year level, described in Section 4.4. Cities are not aggregated into provinces or regions and years are not grouped into periods, so every coefficient is identified from variation within cities over time once the national path in each year has been removed. The heterogeneity analysis in Section 6.2 follows the same principle: the sample is split and the same specification is re-estimated on each subsample, rather than observations being combined, which is why the subsample observation counts in Tables 2, 3, 4 sum to the full sample.

**Table 2:** Regional heterogeneity tests.

Variables	**(1)**	**(2)**	**(3)**	**(4)**
	Eastern	Central	Western	Northeastern
Airq	−0.526***	−0.608***	−0.246**	−0.255
	(−4.398)	(−5.251)	(−2.169)	(−1.109)
RGDP	0.000***	0.000***	0.000***	−0.000***
	(7.812)	(2.902)	(2.741)	(−2.937)
Nid	0.000	−0.000	0.000	−0.001***
	(0.503)	(−1.237)	(1.351)	(−5.876)
Nfdi	0.001**	−0.005**	−0.015***	0.003**
	(2.323)	(−2.013)	(−4.620)	(2.134)
Tue	0.000*	0.000***	0.000***	0.000***
	(1.926)	(5.929)	(8.472)	(3.765)
Shoh	−0.001***	−0.000	−0.001***	0.001
	(−2.812)	(−0.713)	(−4.135)	(0.899)
Mpu	0.000	0.002***	0.001***	0.003***
	(1.006)	(5.056)	(3.590)	(5.752)
Npe	0.000	−0.000***	−0.000***	0.000**
	(0.596)	(−4.981)	(−6.972)	(2.274)
Tsoe	−0.000***	−0.000***	−0.000***	−0.000***
	(−9.534)	(−6.239)	(−5.264)	(−2.838)
City FE	YES	YES	YES	YES
Year FE	YES	YES	YES	YES
Constant	10.295***	10.325***	10.590***	9.458***
	(52.824)	(64.609)	(74.688)	(24.202)
Observations	1144	1040	1066	351
*R*-squared	0.504	0.602	0.614	0.720
Number of id	88	80	82	27

*t*-statistics in parentheses. ***p < 0.01, **p < 0.05, *p < 0.1.

**Table 3:** Population mobility heterogeneity tests.

Variables	**(1)**	**(2)**
	Net inflow	Net outflow
Airq	−0.342***	−0.642***
	(−2.822)	(−8.039)
RGDP	0.000***	0.000
	(7.306)	(1.628)
Nid	−0.000	−0.000
	(−1.097)	(−1.347)
Nfdi	0.000	−0.005***
	(1.111)	(−4.114)
Tue	0.000	0.000***
	(1.000)	(9.140)
Shoh	−0.000	−0.001***
	(−0.291)	(−5.405)
Mpu	0.000**	0.001***
	(2.391)	(3.557)
Npe	0.000	−0.000
	(0.621)	(−0.710)
Tsoe	−0.000***	−0.000***
	(−11.429)	(−8.885)
City FE	YES	YES
Year FE	YES	YES
Constant	10.139***	10.766***
	(49.014)	(113.976)
Observations	1274	2327
*R*-squared	0.489	0.562
Number of id	98	179

*t*-statistics in parentheses. ***p < 0.01, **p < 0.05, *p < 0.1.

**Table 4:** Urban resource heterogeneity tests.

Variables	**(1)**	**(2)**	**(3)**	**(4)**	**(5)**
	Resource-based				Non-resource
	Growth	Mature	Declining	Regenerating	
Airq	−0.764**	−1.011***	−0.784***	−0.284**	−0.356***
	(−2.002)	(−8.447)	(−4.550)	(−2.126)	(−3.764)
RGDP	−0.000**	−0.000	0.000	0.000	0.000***
	(−2.168)	(−0.989)	(0.062)	(0.257)	(8.983)
Nid	0.001**	−0.000***	0.000**	−0.000*	−0.000*
	(2.011)	(−3.525)	(1.999)	(−1.827)	(−1.715)
Nfdi	−0.000	−0.006***	−0.007*	0.004*	0.001***
	(−0.005)	(−3.426)	(−1.936)	(1.683)	(3.539)
Tue	0.000***	0.000***	0.000***	0.000	0.000**
	(4.101)	(8.255)	(2.724)	(1.649)	(2.074)
Shoh	−0.001***	0.000	−0.000	0.000	−0.001***
	(−3.196)	(0.236)	(−0.403)	(0.060)	(−4.819)
Mpu	−0.001	0.001*	0.000	0.000	0.000***
	(−0.884)	(1.813)	(0.228)	(0.316)	(3.391)
Npe	−0.000	−0.000**	−0.000	−0.000	0.000
	(−0.286)	(−2.261)	(−0.605)	(−1.195)	(0.037)
Tsoe	−0.000	−0.000***	−0.000***	−0.000***	−0.000***
	(−0.191)	(−5.618)	(−7.341)	(−7.573)	(−13.697)
City FE	YES	YES	YES	YES	YES
Year FE	YES	YES	YES	YES	YES
Constant	11.585***	11.170***	11.126***	11.007***	10.306***
	(27.539)	(68.400)	(43.881)	(38.887)	(76.873)
Observations	286	559	390	286	2080
*R*-squared	0.538	0.665	0.721	0.751	0.500
Number of id	22	43	30	22	160

*t*-statistics in parentheses. ***p < 0.01, **p < 0.05, *p < 0.1.

### 4.2 Variable selection

#### 4.2.1 Dependent variable

Public Attention to Depressive Emotions (Dpre). Drawing on the research paradigm of internet search behavior analysis, this study adopts the Baidu Index as the core data source. The Baidu Index is a data analysis platform based on massive user behavioral data from the Baidu search engine, capable of reflecting the search intensity of specific keywords within specific time periods and geographic ranges, and has been widely used to track dynamic trends in public health, socioeconomic conditions, and public sentiment (Liang et al., 2025). Regarding keyword selection:

First, based on conceptual direct relevance and common expressions in online searches, three core keywords were selected: “depression” (basic emotional expression), “depressive disorder” (clinical disease cognition), and “what are the symptoms of depression” (active self-examination and information exploration). These three keywords, respectively, correspond to a continuous spectrum of public attention from general affect to specific disease cognition, possessing good content validity. Second, for each keyword the daily index from 1 January 2011 to 31 December 2023 was retrieved for each prefecture-level administrative region, the finest geographic unit reported by the platform, and aggregated to a city-year total. Finally, the three city-year totals were summed with equal weight and the sum was log-transformed. Because the three keywords differ substantially in base search volume, Table 5 re-estimates the baseline model with each keyword entered separately, so that the findings do not rest on the weighting of the composite.

**Table 5:** Robustness tests with alternative variable measures.

Variables	**(1)**	**(2)**	**(3)**	**(4)**
	Dpre1	Dpre2	Dpre3	Dpre
Airq / Airq2	−0.166***	−0.717***	−2.340***	−0.001***
	(−4.830)	(−5.550)	(−5.279)	(−3.757)
RGDP	0.000***	0.000***	0.000	0.000***
	(5.920)	(4.139)	(1.217)	(5.173)
Nid	0.000*	−0.000***	−0.000	−0.000
	(1.836)	(−3.232)	(−0.073)	(−0.537)
Nfdi	0.001	−0.001**	0.000	0.002***
	(1.458)	(−2.250)	(0.236)	(3.544)
Tue	0.000	0.000***	0.000	0.000***
	(0.743)	(3.590)	(1.119)	(5.132)
Shoh	−0.002***	−0.001***	−0.002***	−0.001***
	(−3.555)	(−2.818)	(−3.063)	(−8.802)
Mpu	0.001**	0.000	−0.001	0.001***
	(2.265)	(0.952)	(−1.387)	(3.478)
Npe	−0.000	−0.000	−0.000	−0.003
	(−1.459)	(−1.009)	(−0.136)	(−1.110)
Tsoe	−0.000***	−0.000***	−0.000*	−0.001***
	(−7.261)	(−7.825)	(−1.762)	(−12.657)
City FE	YES	YES	YES	YES
Year FE	YES	YES	YES	YES
Constant	7.020***	11.013***	7.518***	10.602***
	(22.424)	(58.489)	(12.207)	(10.574)
Observations	3601	3601	3601	3601
*R*-squared	0.467	0.478	0.158	0.443
Number of id	277	277	277	277

*t*-statistics in parentheses. ***p < 0.01, **p < 0.05, *p < 0.1.

Meanwhile, to verify the validity of the Baidu Index as a proxy indicator for public attention to depressive emotions, this study provides validity evidence from the following three aspects.

First, content validity: The three keywords selected in this study (“depression,” “depressive disorder,” and “what are the symptoms of depression”) cover a complete spectrum from emotional expression to disease cognition to active help-seeking, capable of comprehensively capturing public attention to depression-related topics.

Second, criterion-related validity: This study conducted correlation tests between the constructed Baidu Index depression emotion indicator and provincial psychiatric hospital outpatient visit data from the China Health Statistics Yearbook, revealing a significant positive correlation (*r* = 0.412, *p* < 0.01), indicating that online search behavior can, to a certain extent, reflect actual mental health service demand.

Third, support from existing research: In recent years, using online search data to track public psychological states has become an important method in behavioral epidemiology. Ayers et al. (2013) showed that Google search volume for mental health terms follows systematic seasonal patterns in both the United States and Australia; Liang et al. (2025) further validated the association between Baidu depression search indices and urban built environments. These studies provide methodological support for this study’s measurement strategy.

It should be noted that the Baidu Index reflects public “attention” to depression topics rather than direct “prevalence rates.” However, considering that: (1) information search behavior is often driven by internal psychological needs; (2) individuals experiencing emotional distress tend to seek information and help through the internet; and (3) this indicator can circumvent social desirability effects in traditional surveys, this study considers it reasonable to use this as a proxy indicator for depressive emotions at the population level.

#### 4.2.2 Explanatory variable

Air Quality (Airq). This study uses official monitoring data released by China’s Ministry of Ecology and Environment. Specifically, the Air Quality Index (AQI), calculated under the Technical Regulation on Ambient Air Quality Index (HJ 633–2012) on the basis of China’s Ambient Air Quality Standards (GB 3095–2012), is used as the evaluation basis. AQI is a composite index comprehensively considering six major pollutants—fine particulate matter (PM2.5), inhalable particulate matter (PM10), sulfur dioxide (SO2), nitrogen dioxide (NO2), ozone (O3), and carbon monoxide (CO)—capable of relatively comprehensively reflecting a city’s overall air pollution level.

To characterize a city’s sustained capacity to provide clean air as a basic environmental public good throughout the year, this study did not simply use the annual mean AQI, but instead adopted an indicator with greater policy implications and public perception significance: the proportion of days in a year when air quality meets or exceeds the national Grade II standard. The calculation method is: for each city, classify each day’s AQI by grade throughout the year, count the number of days with AQI values not exceeding 100 (i.e., air quality rated “excellent” or “good”), then divide by the number of days with valid monitoring in that year. Its formula expression is:

Proportion of days meeting air quality standards = Number of days with AQI ≤ 100 annually / Number of days with valid monitoring in that year.

For city-years before full implementation of GB 3095–2012 AQI monitoring, daily API monitoring coverage was limited to key environmental protection cities and could not cover all 277 prefecture-level samples. Therefore, for observations prior to full local AQI reporting, this study directly adopts the annual count of days with air quality at Grade II or better extracted from municipal statistical yearbooks, statistical bulletins and ecological-environment status bulletins. The compliance-day proportion is then computed as the reported annual compliant days divided by valid monitoring days for that city-year. Under both the old API (GB 3095–1996) and new AQI (GB 3095–2012) standards, values ≤100 correspond to air quality at Grade II or better. Section 4.4 discusses the measurement-basis break induced by the monitoring-standard transition.

#### 4.2.3 Mediating variable

Unemployment Rate (Ur). Measured as natural logarithm of annual number of urban registered unemployed persons. Data are sourced from the “Employment, Unemployment and Wages” section of the China Urban Statistical Yearbook for the corresponding years.

#### 4.2.4 Moderating variables

Healthcare Workforce (Nmh). Measured as the natural logarithm of the annual number of licensed (assistant) physicians. Data are sourced from the China Urban Statistical Yearbook. This indicator is fundamental for assessing regional healthcare human resource accessibility and public health service levels.

Urban Public Fiscal Expenditure (Upe). Measured as the natural logarithm of annual local general public budget expenditure (unit: 10,000 yuan). Data are sourced from the “Finance” section of the China Urban Statistical Yearbook.

#### 4.2.5 Control variables

To improve research precision, based on research questions and data availability, and referencing existing literature, eight control variables were selected: (1) Economic development level (per capita regional GDP, RGDP); (2) Industrial development level (number of above-scale industrial enterprises, Nid); (3) Opening-up level (number of foreign-invested enterprises, Nfdi); (4) Total fixed asset investment (annual total fixed asset investment of the whole society, Tue); (5) Healthcare level (sum of hospitals and health centers, Shoh); (6) Information technology level (year-end mobile phone subscribers, Mpu); (7) Unemployment insurance coverage (number of unemployment insurance participants, Npe); (8) Environmental level (industrial sulfur dioxide emissions, Tsoe).

### 4.3 Data sources and descriptive statistics

This study selects 277 prefecture-level and above cities in China as samples, with a time span from 2011 to 2023. The reasons for choosing 2011 as the sample starting year are as follows: First, city-level data from the Baidu Index became relatively complete starting from 2011, with substantial missing data in earlier years; Second, China’s ambient air quality monitoring network was gradually improved starting from 2012, with 2011 data serving as a baseline reference; Third, China’s urbanization process has accelerated since 2011, with urban-level environmental and mental health issues becoming increasingly prominent, possessing strong research value. Except for public attention to depressive emotions indices from the Baidu Index, air quality from official monitoring data released by China’s Ministry of Ecology and Environment (supplemented, for city-years before full AQI reporting, by the officially published compliant-day counts described in Section 4.2), and the meteorological variables used in Table 6, all other data are from corresponding years’ China Urban Statistical Yearbook and city statistical bulletins. To avoid extreme values interfering with empirical results, all continuous variables in the sample were winsorized at the 1 and 99% levels. Descriptive statistics for relevant variables are presented in Table 7:

**Table 6:** Robustness tests controlling for meteorological conditions.

Variables	**(1)**	**(2)**	**(3)**
	Dpre	Dpre	Dpre
Airq	−0.470***	−0.393***	−0.388***
	(−6.757)	(−6.032)	(−5.402)
Sun		−0.013***	−0.010***
		(−4.097)	(−3.779)
Temp			−0.045***
			(−4.301)
Prec			0.071***
			(7.019)
Wind			−0.063***
			(−8.100)
Hum			0.082***
			(4.790)
Controls	YES	YES	YES
City FE	YES	YES	YES
Year FE	YES	YES	YES
Observations	3601	3601	3601
*R*-squared	0.492	0.516	0.559
Number of id	277	277	277

*t*-statistics in parentheses. ***p ≤ 0.01, **p ≤ 0.05, *p ≤ 0.1. Column (1) reproduces Table 9, column (5). Controls are the eight control variables of Table 9, column (5).

**Table 7:** Descriptive statistics.

Variable	*N*	Mean	Std. Dev.	Min	Max
Dpre	3601	10.763	0.753	5.996	13.151
Airq	3601	0.814	0.161	0.071	1.000
Ur	3601	7.879	0.887	5.797	9.037
Nmh	3601	13.387	0.761	10.349	16.323
Upe	3601	13.701	1.514	11.624	18.930
RGDP	3601	64,604.246	35,634.820	10,987.000	243,780.000
Nid	3601	1,519.923	1,861.191	3.000	13,844.000
Nfdi	3601	122.338	339.490	1.000	3,292.000
Tue	3601	5,405,132.706	7,895,780.690	220,571.000	96,385,132.000
Shoh	3601	151.799	126.952	5.000	994.000
Mpu	3601	551.036	562.885	15.000	4,594.000
Tsoe	3601	26,339.654	38,242.663	1.000	404,340.000
Npe	3601	764,446.928	1,491,664.605	11,279.000	14,184,000.000

### 4.4 Temporal granularity and data aggregation

The variables entering the model come from sources with very different native frequencies, so the alignment procedure is set out explicitly. The Ministry of Ecology and Environment publishes AQI at hourly and at daily frequency; this study uses neither the hourly readings nor the annual mean. For city-years with complete daily AQI monitoring under HJ 633–2012, each daily official AQI value is graded; days with AQI ≤ 100 are counted as compliant, and the annual compliant-day proportion equals compliant-day count divided by valid monitoring days in the calendar year. For city-years before full local AQI reporting, we use the officially published annual number of days meeting the Grade II standard from yearbooks and environmental bulletins to construct the same proportion indicator. The Baidu Index is retrieved at daily frequency for each of the three keywords and for each prefecture-level administrative region, and daily values are summed to city-year totals before the three totals are added and log-transformed. Every remaining variable is annual by construction, since it originates in the China Urban Statistical Yearbook.

No series was interpolated or upsampled. Alignment ran in the opposite direction, aggregating the two high-frequency series down to the annual level, which is the coarsest frequency common to all sources. The choice is deliberate rather than merely convenient. Disaggregating annual yearbook variables such as unemployment or fiscal expenditure to monthly or daily values would require assumptions about within-year paths that the data cannot support, and it would generate serial correlation that is an artifact of the interpolation rule rather than a property of the data. Aggregating upward discards within-year variation but introduces no such artifact, and it matches the object of interest, since the question asked here concerns a city’s sustained capacity to supply clean air across a year rather than its response to individual pollution episodes. The unit of observation throughout is the city-year and the panel is balanced.

One feature of the air quality series should be flagged. China’s move from the API to the AQI system was phased rather than simultaneous. GB 3095–2012 was issued in 2012; real-time AQI reporting under it began in 74 key cities on 1 January 2013, coverage was extended to 161 cities on 1 January 2014 and to all prefecture-level and above cities from 1 January 2015, and the standard took effect nationwide on 1 January 2016. The monitoring network expanded over the same period, so compliance shares before and after the transition are not measured on an identical basis, and Figure 2 makes the resulting break visible. Year fixed effects absorb the component of this break that is common to all cities, and identification rests on within-city deviations from the national path in a given year rather than on the level of the series. Because cities switched in different years, however, year fixed effects do not absorb city-specific differences in the timing of the switch. To verify that this timing does not drive the results, the baseline specification is re-estimated on 2015–2023, the window in which every sample city reports AQI under GB 3095–2012 (2,493 observations); the coefficient on air quality is −0.403 [*t* = −6.004; Table 8, column (3)], compared with −0.470 in the full sample, and remains significant at the 1% level.

**Table 8:** Sample adjustment robustness tests.

Variables	**(1)**	**(2)**	**(3)**
	Dpre	Dpre	Dpre
Airq	−0.478***	−0.495***	−0.403***
	(−5.720)	(−7.375)	(−6.004)
RGDP	0.000***	0.000***	0.000***
	(10.423)	(4.864)	(7.662)
Nid	−0.000**	−0.000***	−0.000***
	(−2.499)	(−4.537)	(−5.318)
Nfdi	0.000	−0.001***	−0.001***
	(1.517)	(−3.766)	(−9.404)
Tue	0.000**	0.000***	0.000***
	(2.484)	(10.704)	(9.228)
Shoh	−0.001***	−0.001***	−0.001***
	(−4.824)	(−5.988)	(−4.907)
Mpu	0.001***	0.001***	0.001***
	(5.751)	(8.172)	(7.070)
Npe	−0.000	−0.000***	−0.000***
	(−1.534)	(−6.108)	(−5.411)
Tsoe	−0.000***	−0.000***	−0.000***
	(−13.940)	(−12.851)	(−14.912)
City FE	YES	YES	YES
Year FE	YES	YES	YES
Constant	10.332***	10.614***	10.004***
	(83.205)	(117.800)	(78.575)
Observations	2770	3549	2493
*R*-squared	0.529	0.541	0.398
Number of id	277	273	277

*t*-statistics in parentheses. ***p < 0.01, **p < 0.05, *p < 0.1. Column (1) excludes 2020–2022, column (2) excludes the four municipalities, and column (3) restricts the sample to 2015–2023, when every sample city reports AQI under GB 3095–2012.

### 4.5 Temporal and regional patterns

Table 7 summarizes variation in the pooled sample but says nothing about its shape over time or across space. Figure 2 plots the two core variables for the 2011–2023 window, in aggregate and by region, for the 120 cities with continuous air quality monitoring records in every year.

Panel (a) gives the aggregate picture. Air quality improves from its 2014 low, though not monotonically: the mean compliant-day share rises from 0.674 in 2014 to a peak in 2021 and stands at 0.822 in 2023, while mean annual AQI falls from 94.8 to 74.8. Attention to depressive emotions does not mirror that improvement. It rises through 2019, falls back in 2020, holds roughly level in 2021 and declines thereafter. The two aggregate series accordingly do not co-move in any simple inverse fashion, and the central claim of this paper cannot be read off the aggregate trends. Over this period attention is dominated by forces that operate nationally and have nothing to do with air quality: the spread of mobile internet use, the rising salience and falling stigma of mental illness, and the COVID-19 shock. Those are exactly the influences that year fixed effects remove.

Panels (b) and (c) show that the aggregate conceals substantial and persistent regional spread. Compliant-day shares in 2023 range from 0.777 in the central region to 0.867 in the northeast, and from 2013 onward the central region is the lowest in every year while the northeastern and western regions are the highest, with no sustained narrowing of the spread. Dispersion within regions is in fact larger than dispersion between them. In 2023 the standard deviation of the compliant-day share across cities inside a region averages 0.101, against a range of only 0.090 between the four regional means (the standard deviation of those four means, the like-for-like comparison, is only about 0.04), and city-level shares in that year run from 0.581 to 1.000. Most of the usable cross-sectional variation therefore lies between cities within a region rather than between regions, which is one reason the analysis is conducted at city rather than regional level. Attention is consistently highest in eastern cities and lowest in western cities, a gap that tracks differences in internet penetration and urban scale and that city fixed effects absorb. The two variables do not order the regions in the same way, which again indicates that the relationship of interest is not recoverable from levels.

Taken together the three panels indicate that neither the national trend nor the cross-regional ordering can be used to assess the relationship of interest, and this is worth making explicit because the aggregate series invite the opposite reading. The estimator used below does not compare a polluted year with a clean year nationally, nor a polluted region with a clean one. It compares a city with itself, relative to the national path in the same year. Column (1) of Table 9 reports exactly that comparison with no controls beyond the two sets of fixed effects, and the coefficient is −0.176 and significant at the 5% level. A city whose air quality improves faster than the national path in a given year therefore records a relative fall in attention to depressive emotions, even though the aggregate series in Figure 2 show attention rising over most of the period in which air quality improved.

**Table 9:** Baseline regression results.

Variables	**(1)**	**(2)**	**(3)**	**(4)**	**(5)**
	Dpre	Dpre	Dpre	Dpre	Dpre
Airq	−0.176**	−0.431***	−0.439***	−0.491***	−0.470***
	(−2.152)	(−5.357)	(−5.586)	(−6.530)	(−6.757)
RGDP		0.000***	0.000***	0.000***	0.000***
		(27.983)	(17.898)	(13.043)	(9.118)
Nid		−0.000***	−0.000***	−0.000**	−0.000
		(−2.867)	(−4.082)	(−2.180)	(−1.217)
Nfdi			−0.000	−0.000	0.001***
			(−1.064)	(−0.650)	(2.808)
Tue			0.000***	0.000***	0.000***
			(8.443)	(4.796)	(3.763)
Shoh				−0.001***	−0.001***
				(−10.517)	(−5.943)
Mpu				0.001***	0.000***
				(7.492)	(5.023)
Npe					−0.000
					(−0.864)
Tsoe					−0.000***
					(−18.234)
City FE	YES	YES	YES	YES	YES
Year FE	YES	YES	YES	YES	YES
Constant	10.907***	10.181***	10.282***	10.278***	10.574***
	(162.761)	(138.604)	(124.621)	(105.407)	(114.578)
Observations	3601	3601	3601	3601	3601
*R*-squared	0.112	0.298	0.346	0.406	0.492
Number of id	277	277	277	277	277

*t*-statistics in parentheses. ***p < 0.01, **p < 0.05, *p < 0.1.

## 5 Empirical analysis

### 5.1 Baseline regression

Table 9 reports the baseline regression results of air quality on public attention to depressive emotions. We employed a stepwise approach of progressively adding control variables to examine the robustness of the core explanatory variable (Airq) estimates, with all columns controlling for city and year fixed effects.

The baseline regression results systematically test the relationship between air quality and public attention to depressive emotions. Within the panel model framework controlling for city and year fixed effects, regression results across all models robustly support research hypothesis H1—that the proportion of days meeting air quality standards is significantly negatively correlated with public attention to depressive emotions. Specifically, in Model (1) containing only the core explanatory variable, the estimated coefficient for air quality (Airq) is −0.176, significant at the 5% level. As urban characteristic variables including economic development, industrial scale, openness, investment, health services, information infrastructure, unemployment insurance and emissions are progressively introduced, the absolute value of the air quality coefficient rises from 0.176 to between 0.431 and 0.491, with significance rising to the 1% level. In the complete Model (5) incorporating all control variables, the coefficient for Airq stabilizes at −0.470 (95% confidence interval: −0.606 to −0.334), highly significant at the 1% level. This result indicates that, after controlling for other socioeconomic factors, each one percentage point increase in the proportion of days meeting urban air quality standards corresponds to an average decrease of approximately 0.47% in public attention to depressive emotions. From the trend of coefficient changes, the introduction of control variables not only did not weaken the core relationship but strengthened its robustness, suggesting that omitting urban socioeconomic characteristics may lead to underestimation of air quality’s mental health effects. The above results provide strong empirical support for the proposition that “clean air, as an important environmental public good, plays a fundamental role in maintaining public mental health,” and hypothesis H1 is verified.

Two aspects of the control set deserve comment. The sequence in which controls enter Table 9 does not affect the estimates in the full specification. Least squares with fixed effects is invariant to the order in which regressors are listed; what varies across columns (1) to (5) is the set of controls included, not their ordering, and any other entry sequence reproduces the same coefficients in the corresponding full specification; the intermediate columns would contain different subsets of controls under a different sequence, which is why they are presented as a sensitivity display rather than as alternative estimates. The sequence used here follows a substantive logic, moving from aggregate economic scale, through industrial and openness characteristics and investment, to public service and information infrastructure, and finally to social insurance and environmental pressure. Column (5), containing all eight controls, is the specification carried forward throughout the paper.

Several controls are themselves significantly associated with attention to depressive emotions, and the signs are largely as expected; because these controls enter in levels (yuan, counts and tonnes) rather than in logarithms, their coefficients are 0.000 or ±0.001 at three decimal places, so Table 9 conveys their sign and precision rather than their magnitude. Per capita GDP and mobile phone subscriptions both enter positively, consistent with search activity rising with income and with internet access rather than with mental health deteriorating as cities grow richer. This is a reporting channel, and it is one reason attention should not be read directly as prevalence. The count of hospitals and health centers enters negatively, consistent with better-provisioned cities routing health concerns into offline consultation. Industrial sulfur dioxide emissions also enter negatively once air quality is controlled for, which is initially counterintuitive; the most plausible reading is that, conditional on realized air quality, emission intensity proxies for a manufacturing-oriented employment structure and an older, less online population rather than for additional exposure. What matters for the present argument is that the coefficient on air quality is negative and significant in every column of Table 9 and, once any controls are included, remains between −0.431 and −0.491 and significant at the 1% level, so the finding does not hinge on which of these economic factors are included.

### 5.2 Robustness tests

#### 5.2.1 Alternative measures for dependent and explanatory variables

Regressions were re-estimated using the logarithm of “depression” word frequency (Dpre1), logarithm of “depressive disorder” word frequency (Dpre2), and logarithm of “what are the symptoms of depression” word frequency (Dpre3) to measure public attention to depressive emotions; results are shown in Table 5, columns (1) through (3). Column (4) retains Dpre as the dependent variable and replaces the explanatory variable with the annual count of days meeting or exceeding the Grade II standard (Airq2), so the coefficient reported in the Airq / Airq2 row of that column is the coefficient on Airq2. The estimated coefficients for air quality are all significantly negative, indicating the baseline conclusions are robust.

#### 5.2.2 Lagged effects of core variables

Following standard practice in this literature, the core explanatory variable was lagged by one to three periods and regressions re-estimated; results are shown in Table 1, columns (1) through (3). The impacts of one- to three-period lagged core explanatory variables on public attention to depressive emotions are all significantly negative, indicating that the influence of air quality persists beyond the contemporaneous year: although the magnitude declines from −0.816 at one lag to −0.523 at three lags, all three coefficients remain significant at the 1% level. Air quality can exert cumulative negative effects on public attention to depressive emotions over longer time series, providing indirect support for H1.

#### 5.2.3 Sample adjustment tests

Sample adjustments were made from three aspects: the study window period, municipalities and the measurement basis of air quality. First, the major public health event that emerged at the end of 2019 caused negative shocks to global economic activities and had obvious impacts on public attention to depressive emotions, potentially leading to substantial estimation bias. The concern is specific rather than general. Lockdowns improved measured air quality while simultaneously altering search behavior, so the pandemic years could in principle produce a spurious association for reasons unrelated to the mechanism proposed here. The sign of that association cannot be established in advance: the most direct channel, in which cleaner air coincided with heightened pandemic-related anxiety, would work against the estimated negative effect, while local differences in the timing and stringency of restrictions could operate in either direction. This study therefore re-estimated regressions after excluding the 2020 to 2022 samples; Table 8, column (1) reports the relevant results. After excluding the pandemic years, the coefficient for air quality remains significantly negative at −0.478, against a baseline estimate of −0.470, indicating that the result is not driven by the pandemic period. The nationwide component of the COVID-19 shock is in any case absorbed by the year fixed effects that every specification includes.

Second, considering that municipalities differ substantially from other cities in economic and policy environments, with air quality and public attention to depressive emotions characteristics also potentially differing significantly, this study re-estimated regressions after excluding municipality samples; Table 8, column (2) reports the relevant results. The coefficient for air quality remains significantly negative with little change, demonstrating robust results.

This second test also speaks to the possible influence of the Tianjin Port explosion of August 2015, since Tianjin is one of the four municipalities dropped. The event leaves no detectable trace in annual city-level air quality. Tianjin’s compliant-day share was 0.463 in 2014, 0.609 in 2015 and 0.614 in 2016, and its mean annual AQI was 120.2, 103.7 and 104.1 in those 3 years, so 2015 records an improvement rather than a deterioration on the preceding year. That is what would be expected of a catastrophe which was severe but confined to one district and to a period of days, whereas the measure used here is an annual city-wide aggregate. Dropping Tianjin together with the other municipalities leaves the estimated coefficient at −0.495 and significant at the 1% level.

Third, because AQI reporting under GB 3095–2012 was phased in between 2013 and 2015 (Section 4.4), the sample is restricted to 2015–2023, the window in which every sample city reports under the new standard; Table 8, column (3) reports the results. The coefficient on air quality is −0.403 (*t* = −6.004), significant at the 1% level and of similar magnitude to the full-sample estimate of −0.470, so the phased transition between monitoring standards does not drive the baseline result.

#### 5.2.4 Controlling for meteorological conditions

Weather enters the relationship in two ways. Wind, precipitation and atmospheric stability govern how emissions disperse and carry pollution across city boundaries, while sunshine duration and cloud cover are plausible direct influences on mood, so city-specific weather in a given year could confound the estimate. Annual meteorological variables were therefore constructed for every sample city, namely sunshine duration (Sun), mean temperature (Temp), precipitation (Prec), mean wind speed (Wind) and mean relative humidity (Hum), and added to the baseline specification. Table 6 reports the results. Column (1) reproduces the baseline estimate, column (2) adds sunshine duration, which captures the cloud-cover channel most directly, and column (3) adds all five meteorological variables. The coefficient on air quality is −0.393 (t = −6.032) in column (2) and −0.388 (t = −5.402) in column (3), compared with −0.470 in the baseline, so conditioning on weather attenuates the estimate by about 16 and 17%, respectively, but leaves it negative and significant at the 1% level. Sunshine duration itself enters negatively and significantly in both columns, consistent with a direct affective channel, yet adding it moves the air quality coefficient only modestly, which indicates that the baseline association does not simply proxy for cloud cover. The baseline conclusion is therefore robust to city-specific weather.

#### 5.2.5 Spatially robust inference

The baseline standard errors treat city-year observations as independent conditional on the fixed effects, whereas the regression errors may be serially correlated within cities and, because pollution is transported between neighboring cities and search behavior diffuses through networks that cross administrative boundaries, spatially correlated across cities, so the precision of the estimate may be overstated. Table 10 therefore re-estimates the baseline specification with standard errors clustered at the city level (column 1) and at the province level (column 2), and with Conley (1999) spatial standard errors using distance cut-offs of 200 km and 500 km (columns 3 and 4). The point estimate is identical across columns by construction; the t-statistic on air quality lies between −4.879 (Conley, 500 km) and −6.314 (city clustering), and the coefficient remains significant at the 1% level in every column, so the baseline inference is not an artifact of serial or spatial correlation in the errors.

**Table 10:** Robustness of inference to spatial correlation.

Variables	**(1)**	**(2)**	**(3)**	**(4)**
	Dpre	Dpre	Dpre	Dpre
Airq	−0.470***	−0.470***	−0.470***	−0.470***
	(−6.314)	(−5.478)	(−5.007)	(−4.879)
Standard errors	City cluster	Province cluster	Conley, 200 km	Conley, 500 km
Controls	YES	YES	YES	YES
City FE	YES	YES	YES	YES
Year FE	YES	YES	YES	YES
Observations	3601	3601	3601	3601
Number of id	277	277	277	277

*t*-statistics in parentheses. ***p ≤ 0.01, **p ≤ 0.05, *p ≤ 0.1. All columns estimate the specification of Table 9, column (5), so the coefficients are identical and only the standard errors differ. Columns (3) and (4) use Conley (1999) standard errors, which allow the errors of cities within the stated distance of each other to be correlated.

### 5.3 Endogeneity tests

#### 5.3.1 Instrumental variable estimation

To address potential endogeneity issues from measurement error and omitted variables in the air quality variable, we introduce an instrumental variable for two-stage least squares estimation. Drawing on identification strategies commonly used in atmospheric physics and environmental economics, we select “the average proportion of days meeting annual air quality standards among geographically adjacent cities” as the instrumental variable. For target city i in year t, we calculate the simple arithmetic mean of the proportion of days meeting air quality standards (Airq) in year t for all its geographically adjacent cities (cities sharing borders), denoted as IV_Neighbor_Airq.

Since atmospheric pollutants (especially fine particulate matter PM2.5) exhibit significant regional transport characteristics, a city’s air quality is strongly spatially autocorrelated with surrounding areas’ air quality. Therefore, adjacent cities’ air quality is highly correlated with the focal city’s air quality, satisfying the relevance requirement for instrumental variables. Theoretically, other cities’ air quality primarily affects the focal city’s air quality through atmospheric physical processes (such as cross-regional pollutant transport), and should not directly impact focal city residents’ mental health through channels not mediated by the focal city’s air quality. Although inter-regional economic or social connections exist, this study’s model already controls for main socioeconomic characteristic variables of the focal city (such as per capita GDP, fixed asset investment, and industrial scale) as well as city and year fixed effects, which helps absorb potential confounding from regional common trends or associations. Therefore, this instrumental variable can be considered to satisfy the exclusion restriction. Relevant regression results are shown in Table 11.

**Table 11:** Instrumental variable endogeneity tests.

Variables	**(1)**	**(2)**
	First stage	Second stage
IV_Neighbor_Airq	0.080***	
	(9.631)	
Airq		−1.157***
		(−9.661)
RGDP	0.001***	0.000
	(7.011)	(0.637)
Nid	0.003***	−0.001**
	(8.135)	(−2.022)
Nfdi	−0.005***	0.002**
	(−5.334)	(2.122)
Tue	0.000***	0.000
	(14.209)	(1.121)
Shoh	0.035***	−0.001
	(4.086)	(−1.644)
Mpu	−0.005***	−0.000***
	(−3.952)	(−3.378)
Npe	−0.000***	0.000
	(−4.158)	(1.171)
Tsoe	−0.000***	−0.000***
	(−4.118)	(−9.034)
City FE	YES	YES
Year FE	YES	YES
Constant	10.001***	10.847***
	(124.107)	(167.435)
*F*-statistic	92.83***	
LM statistic		89.437***
Wald *F*-statistic		92.777
Stock–Yogo critical value (10% maximal IV size)		16.38
DWH test χ^2^ (*p*-value)		5.23 (0.022)
*R*-squared	0.561	
Observations	3601	3601
Number of id	277	277

*t*-statistics in parentheses. ***p < 0.01, **p < 0.05, *p < 0.1.

From Table 11, the first-stage F-statistic on the excluded instrument (92.83) far exceeds the conventional threshold of 10, indicating a strong correlation between the instrumental variable and the explanatory variable. The LM statistic is 89.437 and significant at the 1% level, rejecting the null hypothesis of under-identification. The Wald F-statistic is 92.777, exceeding the Stock-Yogo critical value of 16.38 for a 10% maximal IV size (Stock and Yogo, 2005), rejecting the null hypothesis that the instrumental variable is weak. Therefore, the instrumental variable constructed in this study is valid and appropriate. In column (1), the first-stage regression coefficient for the instrumental variable is significantly positive at the 1% level; in column (2), the second-stage regression coefficient for air quality is significantly negative at the 1% level, indicating that after addressing potential endogeneity issues, the impact of air quality on public attention to depressive emotions remains evident, and the baseline conclusions are reliable.

#### 5.3.2 Reverse causality and omitted variables

Two distinct threats are worth separating. Reverse causality would require that public attention to depressive emotions in a city changes that city’s air quality within the same year. A direct channel is hard to construct, though an indirect one is conceivable if deteriorating population mental health reduced economic activity and hence emissions; the bias that would produce is positive, pushing the estimated coefficient toward zero, so it would work against the estimated negative effect rather than in its favor and would make the reported estimate conservative rather than inflated. Three features of the design bear on this. The lag structure in Table 1 is the most informative, since attention in year t cannot influence air quality in years t-1, t-2 or t-3, yet the lagged coefficients remain negative and significant at −0.816, −0.544 and −0.523, and the one-period lag is larger in absolute value than the contemporaneous estimate. The instrument provides a second line of defense, because neighboring cities’ compliance shares are determined by their own emissions and by atmospheric transport rather than by the focal city’s search behavior. The sample adjustment tests provide a third, since the years in which a reverse channel is most plausible are precisely the pandemic years that column (1) of Table 8 excludes.

Omitted-variable bias is the more serious concern, and the fixed effects address only part of it. City fixed effects remove all time-invariant urban characteristics, among them climatic normals, terrain, industrial heritage and cultural attitudes toward mental illness. Year fixed effects remove nationwide shocks, including the common component of the 2013–2016 standard transition, the growth of mobile search and the pandemic. What survives is a city-specific, time-varying confounder that moves with air quality and independently with attention to depressive emotions. Local economic conditions are the obvious candidate, which is why per capita GDP, fixed asset investment, industrial scale and openness are controlled for directly, and why the pattern in Table 9 is informative: adding them moves the coefficient on air quality away from zero rather than toward it, from −0.176 without controls to between −0.431 and −0.491 once any of them are included, which indicates that omitting local economic conditions biases the estimate toward zero rather than inflating it. City-specific weather is the second candidate, since it governs pollutant dispersion and may also affect mood directly; adding sunshine duration and four further meteorological controls (Table 6) moves the coefficient only to −0.388, which remains significant at the 1% level. The instrumental variable estimate carries the strongest evidence on this point: the second-stage coefficient of −1.157 exceeds the fixed effects estimate in absolute value, which is the pattern expected when the fixed effects estimate is attenuated by measurement error in annual compliance shares, and not the pattern expected when it is inflated by an omitted local confounder. We nonetheless treat the instrumental variable results as corroborating the sign and significance of the fixed effects estimate rather than as supplying a preferred point estimate, since two-stage least squares is the less precise of the two and the exclusion restriction cannot be tested with a single instrument.

Two formal tests are reported in addition to the discussion above. The first is the Durbin–Wu–Hausman test (Hausman, 1978) in Table 11, which tests the exogeneity of air quality directly rather than by argument; the statistic is χ^2^ = 5.23 (*p* = 0.022). Since the *p*-value is below 0.05, we reject the null hypothesis that air quality is exogenous at the 5% significance level, indicating that Airq is endogenous and that instrumental variable estimation is warranted. Read together with the instrumented coefficient of −1.157, which is larger in absolute value than the fixed-effects estimate of −0.470, the rejection indicates that the bias in the fixed-effects estimate runs toward zero. The baseline estimate is therefore conservative with respect to magnitude, which is why it is retained as the benchmark while the instrumental variable estimate is used to confirm the sign and significance of the effect. The second, reported in Table 12, is a two-step system GMM estimation (Arellano and Bond, 1991; Blundell and Bond, 1998) in which the lagged dependent variable is added and air quality is instrumented by its own lags. System GMM is informative here for two reasons the fixed-effects specification cannot address on its own: attention to depressive emotions is persistent, so an autoregressive term belongs in the model, and internal instruments do not require the exclusion restriction imposed on the neighboring-city instrument. The Arellano–Bond AR(2) test and the Hansen J test (Hansen, 1982) are reported so that instrument validity can be judged rather than assumed. In Table 12 the coefficient on air quality is −0.433 (t = −5.387), close to the fixed-effects estimate; the AR(2) test (*p* = 0.357) does not detect second-order serial correlation in the differenced errors, and the Hansen test (*p* = 0.203) does not reject the joint validity of the instruments. Taken with the lag structure in Table 1, the sample adjustments in Table 8 and the meteorological controls in Table 6, the design addresses reverse causality and omitted-variable bias with formal statistics as well as with argument.

**Table 12:** Additional endogeneity tests: two-step system GMM.

Variables	**(1)**
	Dpre
L.Dpre	0.837***
	(10.357)
Airq	−0.433***
	(−5.387)
Controls	YES
Year FE	YES
AR(1) *p*-value	0.007
AR(2) *p*-value	0.357
Hansen J *p*-value	0.203
Observations	3324
Number of id	277

*t*-statistics in parentheses. ***p ≤ 0.01, **p ≤ 0.05, *p ≤ 0.1. Observations fall to 3,324 because one lag is used.

## 6 Further analysis

### 6.1 Mechanism analysis

#### 6.1.1 Mediation effect

Based on the theoretical analysis above, to test the mediating effect of unemployment rate, this study follows the three-step mediation effect approach proposed by Baron and Kenny (1986), constructing models as follows:

$$Ur_{\mathrm{it}}=\alpha +\text{βAir}q_{\mathrm{it}}+\rho X_{\mathrm{it}}+\mu_{i}+\lambda_{t}+\varepsilon_{\mathrm{it}}$$ (2)

$$\mathrm{Dpr}e_{\mathrm{it}}=\alpha +\text{βAir}q_{\mathrm{it}}+\mathrm{\gamma U}r_{\mathrm{it}}+\rho X_{\mathrm{it}}+\mu_{i}+\lambda_{t}+\varepsilon_{\mathrm{it}}$$ (3)

where i denotes cities, t denotes years; Dpre represents public attention to depressive emotions; Airq represents air quality; Ur represents unemployment rate; X represents control variables; μ represents city fixed effects, λ represents time fixed effects; ε represents the random error term; α, β, γ, and ρ represent estimated coefficients.

Combining Equations 1, 2, and 3, the stepwise regression method is employed to test the mediating role of unemployment rate. Relevant regression results are shown in Table 13, sequentially presenting the total effect of air quality on public attention to depressive emotions (column 1), the impact of air quality on the mediating variable unemployment rate (column 2), and the direct effect of air quality and the mediating effect of unemployment rate after including the mediating variable (column 3). All models control for a series of urban socioeconomic variables and city and time fixed effects.

**Table 13:** Mediation effects.

Variables	**(1)**	**(2)**	**(3)**
	Dpre	Ur	Dpre
Airq	−0.470***	−0.343***	−0.304***
	(−6.757)	(−7.776)	(−4.339)
Ur			0.485***
			(6.428)
RGDP	0.000***	0.000***	0.000
	(9.118)	(26.828)	(1.560)
Nid	−0.000	0.000***	−0.000**
	(−1.217)	(3.847)	(−2.319)
Nfdi	0.001***	0.001***	0.000
	(2.808)	(5.975)	(1.136)
Tue	0.000***	0.000	0.000***
	(3.763)	(1.222)	(3.491)
Shoh	−0.001***	−0.001***	−0.000***
	(−5.943)	(−7.751)	(−3.893)
Mpu	0.000***	0.000***	0.000***
	(5.023)	(5.215)	(3.618)
Npe	−0.000	−0.000	−0.000
	(−0.864)	(−1.503)	(−0.432)
Tsoe	−0.000***	−0.000***	−0.000***
	(−18.234)	(−8.195)	(−16.353)
City FE	YES	YES	YES
Year FE	YES	YES	YES
Constant	10.574***	8.243***	3.111**
	(114.578)	(62.058)	(2.572)
Observations	3601	3601	3601
*R*-squared	0.492	0.574	0.530
Number of id	277	277	277
Sobel value	4.954 (passed)		

*t*-statistics in parentheses. ***p < 0.01, **p < 0.05, *p < 0.1.

This study employs the stepwise regression method proposed by Baron and Kenny (1986) to test the mediating role of unemployment rate.

Step 1: Testing the total effect. As shown in Table 13, column (1), the regression coefficient of air quality on public attention to depressive emotions is −0.470, significant at the 1% level, indicating that air quality has a significant negative impact on public attention to depressive emotions—better air quality corresponds to lower public attention to depressive emotions. The significant total effect satisfies the prerequisite for mediation effect testing.

Step 2: Testing the impact of the independent variable on the mediating variable. As shown in Table 13, column (2), the regression coefficient of air quality on unemployment rate is −0.343, significant at the 1% level, indicating that air quality has a significant negative impact on unemployment rate—better air quality corresponds to lower urban unemployment rates. This result satisfies the second condition for mediation effect testing.

Step 3: Testing direct and mediation effects. As shown in Table 13, column (3), when both air quality and unemployment rate are included in the model, the regression coefficient of unemployment rate on public attention to depressive emotions is 0.485, significant at the 1% level, indicating that unemployment rate has a significant positive impact on public attention to depressive emotions. Meanwhile, the coefficient for air quality is −0.304, still significant at the 1% level, indicating that after controlling for unemployment rate, the direct effect of air quality on public attention to depressive emotions persists.

Synthesizing the three-step test results—significant total effect, significant impact of independent variable on mediating variable, significant impact of mediating variable on dependent variable with independent variable’s direct effect remaining significant—unemployment rate can be determined to play a partial mediating role between air quality and public attention to depressive emotions. Additionally, the Sobel (1982) test statistic is 4.954 and passes the significance test at the 1% level, further validating the robustness of the mediation effect. Because the Sobel test assumes that the indirect effect is normally distributed, a Monte Carlo confidence interval, which requires only the coefficient estimates and their standard errors (Preacher and Selig, 2012), was also computed. The indirect effect is −0.166 (−0.343 × 0.485) and accounts for 35.4% of the total effect, and the direct and indirect effects (−0.304 and −0.166) sum to the total effect of −0.470 in column (1). The 95% Monte Carlo confidence interval for the indirect effect, based on 2,000,000 draws, is [−0.237, −0.105], and the delta-method interval is [−0.232, −0.101]; both exclude zero. The above results confirm the transmission pathway of “air quality → unemployment rate → public attention to depressive emotions,” and research hypothesis H2 is supported. Regarding the underlying mechanisms, regions with better air quality often possess more advanced industrial structures and stronger economic vitality, capable of attracting quality enterprises and high-caliber talent, creating more employment opportunities, and thereby effectively reducing unemployment rates. Meanwhile, good environmental quality helps enhance comprehensive urban competitiveness, promoting service industry and high-tech industry development, further stabilizing the labor market. Unemployment not only signifies loss of economic income but also accompanies negative consequences including declining social status, loss of self-worth, and reduced social interactions—factors that significantly increase individuals’ risk of developing depressive emotions. Therefore, air quality indirectly maintains public mental health through stabilizing the employment market as a socioeconomic channel.

The mechanism deserves stating with more precision than the coefficients alone convey. The path runs from air quality to the local labor market and only then to psychological outcomes, and each step has its own empirical signature. The first is visible in column (2): a higher compliant-day share is associated with a significantly lower registered urban unemployment rate, the coefficient being −0.343 and significant at the 1% level. The sign and the significance are what the argument requires, and they match the two-sided argument developed earlier, in which polluted air simultaneously reduces effective labor supply through morbidity and absenteeism and reduces labor demand by weakening a city’s ability to retain mobile skilled workers and attract knowledge-intensive employers. The second step is visible in column (3): conditional on air quality, a higher unemployment rate is associated with significantly greater attention to depressive emotions, the coefficient being 0.485 and significant at the 1% level. Because this holds within cities and within years, it is not a comparison between structurally different labor markets but a response to change in a city’s own employment conditions.

What links the two steps psychologically is the breadth of the exposure. A rise in the city unemployment rate is experienced not only by those who lose work but by employed residents who read it as a signal about their own security, and by household members outside the labor force who absorb the material and emotional consequences. That is why a macro rate maps onto an aggregate attention measure rather than onto a count of affected individuals. It is also why the direct effect remains significant at −0.304 once the mediator is included: the labor market carries part of the environmental effect, but the appraisal and support mechanisms set out earlier operate on residents whose employment situation is unchanged, and those mechanisms are not routed through the labor market at all.

#### 6.1.2 Moderation effects

Furthermore, moderation effect models were constructed to analyze the moderating roles of healthcare workforce size and urban public fiscal expenditure in the impact of air quality on public attention to depressive emotions. The specific models are as follows:

$$\begin{array}{l} \mathrm{Dpr}e_{\mathrm{it}}=\alpha +\text{βAir}q_{\mathrm{it}}+\mathrm{\gamma Nm}h_{\mathrm{it}}+\mathrm{\delta Nm}h_{\mathrm{it}}\times \mathrm{Air}q_{\mathrm{it}}+\\ \rho X_{\mathrm{it}}+\mu_{i}+\lambda_{t}+\varepsilon_{\mathrm{it}} \end{array}$$ (4)

$$\begin{array}{l} \mathrm{Dpr}e_{\mathrm{it}}=\alpha +\text{βAir}q_{\mathrm{it}}+\mathrm{\gamma Up}e_{\mathrm{it}}+\mathrm{\delta Up}e_{\mathrm{it}}\times \mathrm{Air}q_{\mathrm{it}}+\\ \rho X_{\mathrm{it}}+\mu_{i}+\lambda_{t}+\varepsilon_{\mathrm{it}} \end{array}$$ (5)

where Nmh represents healthcare workforce size, Upe represents urban public fiscal expenditure, γ and δ represent estimated coefficients, and other variables are defined as before. Equation 4 adds healthcare workforce size and its interaction term with air quality (Nmh × Airq) to the baseline model; Equation 5 adds urban public fiscal expenditure and its interaction term with air quality (Upe × Airq) to the baseline model.

Table 14, column (2) reports the test results for the moderating effect of healthcare workforce size. The main effect coefficient for air quality is −12.164 (*p* < 0.01), and the interaction term coefficient between healthcare workforce size and air quality is 0.868 (*p* < 0.01). Since the interaction term coefficient is positive, this means that as healthcare workforce size increases, the negative impact of air quality on public attention to depressive emotions weakens. In other words, in cities with adequate healthcare workforce allocation, the impact of air quality deterioration on public mental health can be buffered to some extent. Hypothesis H3 is therefore supported in its buffering form: healthcare provision attenuates the environmental effect rather than amplifying it. This is worth stating explicitly, because the two mechanisms proposed above carry opposite predictions. The health assurance mechanism implies attenuation, since institutional protection reduces the health anxiety through which pollution is converted into distress. The resource complementarity mechanism implies amplification, since clean air and healthcare would then be complements in producing health and the marginal psychological return to cleaner air would be larger where healthcare is better. The positive interaction coefficient indicates that the first mechanism dominates over the range of provision observed in these data, and the complementarity channel receives no support here. One clarification is needed for the magnitude. Because the interaction term is entered in levels, the main effect of −12.164 is the estimated effect of air quality when Nmh equals zero, which lies outside the observed range of healthcare provision and is not the quantity to be compared with the baseline. Evaluated at the sample mean of Nmh (13.387), the marginal effect of air quality is −0.544 (−12.164 ± 0.868 × 13.387), and it is that figure, rather than the uncentered main effect, that is comparable with the baseline estimate of −0.470. The linear interaction also implies that the marginal effect reaches zero at an Nmh value of about 14.01 (12.164/0.868), which lies inside the observed range of 10.349 to 16.323. Implied effects above that value turn positive, but they depend on the linear form of the interaction and should not be read as evidence that cleaner air raises attention to depressive emotions in the best-provisioned cities without a specification that relaxes that form.

**Table 14:** Moderation effects.

Variables	**(1)**	**(2)**	**(3)**
	Dpre	Dpre	Dpre
Airq	−0.470***	−12.164***	−0.952*
	(−6.757)	(−17.731)	(−1.787)
Nmh		−0.000	
		(−0.040)	
Nmh*Airq		0.868***	
		(17.087)	
Upe			−0.094**
			(−2.193)
Upe*Airq			0.043***
			(3.890)
RGDP	0.000***	0.000	0.000***
	(9.118)	(1.100)	(6.153)
Nid	−0.000	−0.000***	−0.000**
	(−1.217)	(−4.587)	(−2.180)
Nfdi	0.001***	−0.000	0.000
	(2.808)	(−0.490)	(1.537)
Tue	0.000***	0.000**	0.000***
	(3.763)	(2.240)	(2.997)
Shoh	−0.001***	−0.000***	−0.001***
	(−5.943)	(−4.388)	(−5.396)
Mpu	0.000***	0.000***	0.000***
	(5.023)	(3.520)	(4.655)
Npe	−0.000	−0.000**	−0.000
	(−0.864)	(−2.128)	(−0.865)
Tsoe	−0.000***	−0.000***	−0.000***
	(−18.234)	(−13.297)	(−17.945)
City FE	YES	YES	YES
Year FE	YES	YES	YES
Constant	10.574***	11.224***	9.774***
	(114.578)	(120.857)	(20.750)
Observations	3601	3601	3601
*R*-squared	0.492	0.565	0.510
Number of id	277	277	277

*t*-statistics in parentheses. ***p < 0.01, **p < 0.05, *p < 0.1.

Table 14, column (3) reports the test results for the moderating effect of urban public fiscal expenditure. Results show that the interaction term coefficient between public fiscal expenditure and air quality is 0.043, significantly positive at the 1% level, similarly indicating that public fiscal expenditure attenuates the relationship between air quality and public attention to depressive emotions—as fiscal expenditure levels increase, the negative impact of air quality on public attention to depressive emotions weakens. Evaluated at the sample mean of Upe (13.701), the marginal effect of air quality is −0.363 (−0.952 ± 0.043 × 13.701), and because the implied turning point (0.952/0.043 ≈ 22.1) lies well above the sample maximum of 18.930, the marginal effect remains negative throughout the observed range of fiscal expenditure, from −0.452 at the sample minimum to −0.138 at the sample maximum. Regarding the underlying mechanisms, higher public fiscal expenditure means local governments invest more resources in public services, social security, and infrastructure construction, which can effectively improve residents’ living environments and welfare levels, enhance their capacity to cope with external environmental pressures, and thereby buffer the impact of air pollution on public mental health. Research hypothesis H4 is verified.

In summary, both healthcare workforce size and urban public fiscal expenditure significantly attenuate the process through which air quality affects public attention to depressive emotions. In each case the interaction term is positive while the main effect is negative, so the environmental effect is weaker where public resources are more abundant. This finding reveals the important buffering function of public resource allocation in transmitting environmental health effects, providing empirical evidence for governments to mitigate the mental health harms of environmental pollution through optimizing healthcare resource distribution and increasing public fiscal investment.

### 6.2 Heterogeneity analysis

#### 6.2.1 Regional heterogeneity

To examine regional differences in the impact of air quality on public attention to depressive emotions, this study divides sample cities into eastern, central, western, and northeastern regions according to the standard regional classification method of China’s National Bureau of Statistics. This classification not only reflects the gradient pattern of China’s macroeconomic development (eastern coastal leading development, central rising, western development, northeastern revitalization) but also encompasses systematic differences in industrial structure, environmental regulatory intensity, public service levels, and sociocultural characteristics. Conducting heterogeneity tests based on this regional framework helps reveal the different socioeconomic contexts and institutional environments on which environmental psychological effects depend. Specific regression results are shown in Table 2:

Table 2 reports regional heterogeneity test results for the impact of air quality on public attention to depressive emotions. From the regression coefficients, the impact of air quality on public attention to depressive emotions exhibits significantly differentiated characteristics across regions.

Specifically, the regression coefficient for air quality in the eastern region is −0.526, and −0.608 in the central region, both significant at the 1% level; the western region coefficient is −0.246, significant at the 5% level; the northeastern region coefficient is −0.255, not passing the significance test. In terms of impact intensity, the negative impact of air quality on public attention to depressive emotions is most pronounced in the central region, followed by the eastern region, relatively weaker in the western region, and not statistically significant in the northeastern region. Tests for the equality of coefficients across independent subsamples (Paternoster et al., 1998) qualify this ordering: the central-region coefficient is significantly larger in magnitude than the western one (z = −2.23, *p* = 0.026), the eastern coefficient exceeds the western one only at the 10% level (z = −1.70, *p* = 0.089), and the eastern and central coefficients do not differ from each other (*p* = 0.622). The northeastern point estimate is almost identical to the western one (−0.255 against −0.246, *p* = 0.972) and does not differ significantly from that of any other region, so its lack of significance is at least partly a matter of precision on 27 cities.

Regarding the underlying mechanisms, these regional differences can be explained from the following aspects. The central region is in a stage of rapid industrialization and urbanization advancement, with tensions between economic development and environmental protection being quite prominent; the public is more sensitive to air quality changes, and environmental quality fluctuations therefore have more significant impacts on their mental health. The eastern region has higher economic development levels, with generally higher education levels and health awareness among residents, holding higher expectations and requirements for environmental quality; the psychological gap effect from air pollution is relatively pronounced. The western region’s economic development is relatively lagging, with residents possibly more concerned with satisfying basic survival needs, resulting in relatively weaker psychological responses to environmental quality changes. The lack of statistical significance for air quality’s impact in the northeastern region may stem from the following reasons: First, the relatively small sample size (only 27 cities, 351 observations) limits statistical power; Second, the northeastern region is undergoing deep economic structural transformation and population outflow, with socioeconomic problems such as employment pressure, income decline, and public service contraction that residents face possibly having more dominant impacts on mental health, comparatively weakening the marginal effect of air quality; Third, the northeastern region has long winters with concentrated heating periods, with residents having strong psychological expectations and adaptability for winter air quality decline, possibly reducing their emotional sensitivity to air pollution. This result also suggests that in regions with greater socioeconomic pressure, environmental factors’ impacts on mental health may take a secondary position; future research can further explore the interactive effects of environmental and socioeconomic factors.

The above regional heterogeneity results indicate that the effects of air quality on public mental health are embedded in specific regional development stages and socioeconomic contexts. This finding provides important empirical evidence for formulating differentiated environmental governance and mental health intervention policies.

#### 6.2.2 Population mobility heterogeneity

To further explore group heterogeneity in air quality’s psychological effects, this study conducts grouped tests based on cities’ population mobility directions. Specifically, using each city’s average annual net population migration rate during the sample period as the basis, cities are classified into “net population inflow cities” and “net population outflow cities.” This classification aims to capture systematic differences between these two types of cities in development momentum, population structure, and socioeconomic pressure. Net population inflow cities typically represent economically vibrant regions with abundant employment opportunities, attracting large numbers of migrant workers; while net population outflow cities often face challenges of sluggish economic growth and shrinking local employment opportunities, with youth labor outflow possibly exacerbating local socioeconomic pressures. Such structural differences may significantly moderate residents’ sensitivity and psychological response patterns to environmental quality. Specific results are shown in Table 3:

Table 3 reports heterogeneity test results based on population mobility direction. From the regression coefficients, the impact of air quality on public attention to depressive emotions exhibits significant differences between net population inflow and outflow cities. Specifically, the regression coefficient for air quality in net population inflow cities is −0.342, while for net population outflow cities it is −0.642, both significant at the 1% level. In terms of impact intensity, the negative impact of air quality on public attention to depressive emotions in net population outflow cities is notably stronger than in net population inflow cities, with the former’s absolute coefficient value approximately 1.88 times the latter’s. The difference between the two coefficients is statistically significant (z = −2.07, *p* = 0.039).

Regarding the underlying mechanisms, net population outflow cities often face development predicaments such as insufficient economic growth momentum, singular industrial structures, and limited employment opportunities. The continuous outflow of young and middle-aged labor force further weakens local social vitality and development potential, with remaining residents bearing greater economic pressure and social-psychological burdens. Against this background, air quality deterioration may become the “last straw” exacerbating their negative emotions, making residents in such cities exhibit higher psychological sensitivity to environmental quality changes. In contrast, net population inflow cities typically possess strong economic vitality and diversified employment opportunities; comprehensive public service systems and rich social support networks can buffer the impact of environmental pollution on residents’ mental health to some extent, thereby weakening the impact of air quality changes on public attention to depressive emotions.

The above results indicate that population mobility patterns reflecting urban development trajectories have significant moderating effects on air quality’s mental health effects, with residents of net population outflow cities being more vulnerable to environmental quality changes. This finding suggests that in advancing environmental governance, attention should be focused on mental health needs of residents in population-losing areas, organically combining environmental improvement with social support system construction to effectively alleviate mental health risks in such areas.

#### 6.2.3 Urban resource heterogeneity

To further examine heterogeneity in air quality’s psychological effects across cities with different development trajectories, this study classifies sample cities into resource-based and non-resource-based categories according to the classification standards of the National Development and Reform Commission’s National Sustainable Development Plan for Resource-Based Cities. Resource-based cities are further subdivided into growth-stage, mature-stage, declining-stage, and regenerating-stage types based on their development stages and differing challenges faced. This classification aims to capture unique socioeconomic forms and environmental pressures shaped by resource endowments and industrial life cycles. Different types of resource-based cities have systematic differences in industrial structure, fiscal health status, environmental historical debts, and transformation pressures—structural characteristics that may profoundly moderate residents’ perceptions of and psychological responses to environmental quality. Specific results are shown in Table 4:

Table 4 reports heterogeneity test results based on urban resource endowment characteristics. From the regression coefficients, the impact of air quality on public attention to depressive emotions exhibits significant heterogeneity across different types of cities.

Specifically, within resource-based cities, the regression coefficient for air quality in mature-stage cities is −1.011, significant at the 1% level, with the most pronounced impact intensity; declining-stage cities have a coefficient of −0.784, growth-stage cities −0.764, both passing significance tests; regenerating-stage cities have a coefficient of −0.284, with relatively weaker impact. Non-resource-based cities have a coefficient of −0.356, significant at the 1% level. In terms of impact intensity, a gradient pattern of “mature > declining > growth > non-resource > regenerating” emerges. Tests for the equality of coefficients show which parts of this ordering are statistically reliable: the mature-stage and declining-stage coefficients are each significantly larger in magnitude than those of non-resource cities (*z* = −4.29, *p* ≤ 0.001; *z* = −2.18, *p* = 0.029) and of regenerating-stage cities (*z* = −4.05, *p* ≤ 0.001; *z* = −2.29, *p* = 0.022), whereas mature-stage and declining-stage cities do not differ from each other (*p* = 0.279), and the growth-stage estimate, based on 22 cities, is too imprecise to be distinguished from any other group.

Regarding the underlying mechanisms, these differences can be explained by each city type’s development stage and industrial characteristics. Mature-stage resource cities are in the peak period of resource exploitation, with highly concentrated extractive and heavy chemical industries, prominent environmental pollution problems, and residents’ long-term exposure to higher environmental risks, resulting in the strongest psychological sensitivity to air quality changes. Declining-stage resource cities face multiple pressures including resource depletion, industrial contraction, and economic downturn; residents must cope with historical environmental legacy issues while bearing transformation pains, making air pollution easily become an important trigger for negative emotions. Although growth-stage resource cities are in ascending development phases, the rapid intensification of resource exploitation is often accompanied by simultaneous environmental quality deterioration, with residents being more perceptive to environmental changes. Regenerating-stage resource cities have essentially completed industrial transformation and upgrading, with economic structures trending toward diversification and environmental governance achieving notable results; residents’ psychological states are relatively stable with lower sensitivity to air quality changes. Non-resource-based cities have relatively balanced industrial structures with more dispersed environmental pressures, and residents’ psychological responses are at moderate levels.

The above results indicate that urban resource endowment characteristics and development stages have significant moderating effects on air quality’s mental health effects. Residents of cities in resource exploitation peak periods and transformation difficulty periods are more susceptible to environmental quality fluctuations in their mental health. This finding provides important policy references for green transformation and mental health protection of resource-based cities, suggesting that policymakers should integrate environmental governance with mental health service system construction in advancing sustainable development of resource-based cities.

## 7 Conclusion and policy recommendations

Air pollution is an important environmental issue affecting global public health, and its relationship with mental health has attracted increasing academic attention. To explore the impact of urban air quality on public psychological well-being in China, this study utilizes panel data from 277 prefecture-level cities spanning 2011–2023, measuring environmental quality levels by the annual proportion of days meeting urban air quality standards and constructing public attention to depressive emotions indicators using the “Baidu Index,” breaking through traditional survey methods’ limitations in sample size and timeliness. On this basis, this study incorporates macro socioeconomic variables such as unemployment rates, healthcare workforce allocation, and public fiscal expenditure into the analytical framework, systematically examining the mechanisms through which air quality affects public attention to depressive emotions, and identifying heterogeneity characteristics across multiple dimensions including regional development stages, population mobility patterns, and urban resource endowments. The main conclusions are as follows:

First, air quality improvement has a significant positive effect on alleviating public attention to depressive emotions. Baseline regression results show that increases in the proportion of urban days meeting air quality standards can effectively reduce public online attention to depressive emotions, a conclusion that remains valid after multiple robustness tests including alternative variable measures, consideration of lagged effects, sample adjustments, controls for meteorological conditions, spatially robust inference, instrumental variable estimation and system GMM estimation. These results demonstrate that clean air, as a fundamental environmental public good, plays an irreplaceable role in maintaining public mental health.

Second, the impact of air quality on public attention to depressive emotions is embedded in complex socioeconomic systems, with both indirect transmission pathways and moderation by public resource allocation. Regarding transmission mechanisms, unemployment rate constitutes an important mediating channel—good air quality can enhance urban economic attractiveness, stabilize employment conditions, and thereby alleviate social-psychological pressure arising from accumulated unemployment risks, meaning environmental governance benefits can extend through the labor market to the mental health dimension. Regarding boundary conditions, healthcare workforce allocation and public fiscal expenditure play significant moderating roles: adequate healthcare resources provide institutional protection for residents against environmental health risks, while sufficient fiscal investment enhances residents’ capacity to resist external pressures through improving public service systems—both effectively buffering the negative psychological impacts of air pollution.

Third, the mental health effects of air quality exhibit significant structural differentiation across different types of cities. From the regional dimension, the effect is strongest in the central and eastern regions, whose coefficients do not differ significantly from each other, weaker in the western region, and imprecisely estimated and not statistically significant in the northeastern region. From population mobility patterns, residents of net population outflow cities are more sensitive to environmental quality changes in their psychological states. From resource endowment characteristics, residents of mature-stage cities in peak resource exploitation and declining-stage cities facing transformation difficulties are particularly affected by air quality fluctuations in their mental health.

Based on the above conclusions, this study proposes the following policy recommendations:

First, incorporate air quality governance into the overall strategic framework of public mental health promotion. Given clean air’s fundamental supporting role for public psychological well-being, governments at all levels should break from the traditional mindset of positioning air pollution control solely within the ecological environment domain, viewing it as a foundational measure for maintaining social-psychological health. When formulating environmental governance targets, beyond focusing on technical indicators such as pollutant concentrations, resident mental health improvement should be incorporated into the policy effectiveness evaluation system, promoting the formation of synergy mechanisms between environmental governance and mental health promotion.

Second, coordinate environmental governance and employment protection to fully release the social benefits of ecological improvement. Targeting the transmission pathway through which air quality affects public emotions via the labor market channel, development models mutually promoting environmentally friendly industries and high-quality employment should be actively cultivated. On one hand, enhance cities’ attractiveness to quality enterprises and high-caliber talent through improving regional environmental quality; on the other hand, ensure employment support during industrial green transformation to avoid localized unemployment risks from strengthened environmental regulations, ensuring that environmental improvement’s economic and social benefits reach the broad workforce. Simultaneously, strengthen the buffering and protective functions of healthcare resource allocation and public fiscal investment against environmental health risks, focusing on improving grassroots healthcare service accessibility, enhancing mental health professional allocation, and optimizing public fiscal expenditure structure to construct multi-level social support systems for residents.

Third, implement differentiated environmental mental health intervention strategies. Targeting the urban heterogeneity of air quality’s psychological effects, tailored policy combinations should be adopted for different types of cities. For the central and eastern regions, where the estimated effects are largest, advance environmental governance and mental health service system construction simultaneously, particularly in central cities that are still accelerating industrialization. For net population outflow cities, organically combine environmental improvement with revitalization development and social support network reconstruction to alleviate multiple psychological pressures on remaining residents. For mature-stage and declining-stage resource cities, pay high attention to residents’ psychological adaptation issues during green transformation, reducing the superimposed impact of environmental and economic dual pressures on residents’ mental health through improving social security and psychological assistance mechanisms during transition periods.

## 8 Limitations and future research

Three limitations should be acknowledged, each pointing to a specific extension.

The first concerns meteorology and temporal resolution. Weather enters the relationship twice. It determines measured air quality, since wind speed and direction, boundary-layer height, precipitation and temperature inversions govern how a given emission load disperses and routinely carry pollution across city boundaries, and it is a plausible direct influence on mood, since sunshine duration and cloud cover are associated with seasonal variation in depressive symptoms. Table 6 addresses this concern with annual city-level controls for sunshine duration, temperature, precipitation, wind speed and relative humidity, and the coefficient on air quality remains negative and significant at the 1% level. Annual aggregates cannot, however, capture the within-year coincidence of pollution episodes with particular weather conditions, and they omit boundary-layer height and inversion frequency, so the atmospheric dispersion channel and the direct affective channel are not fully separated. Matching daily air quality, weather and search data, and exploiting plausibly exogenous meteorological variation such as thermal inversions as an instrument, would help separate the two channels and is the most valuable extension of the present design.

The second concerns spatial structure. Both variables are spatially dependent: pollution is transported between neighboring cities, and search behavior diffuses through media and social networks that follow regional rather than administrative boundaries. Table 10 shows that inference is robust to this dependence, since the coefficient on air quality remains significant at the 1% level under city-clustered, province-clustered and Conley standard errors, and three further pieces of evidence indicate that the city-level results are consistent with the regional picture. The instrument is built from adjacent cities and is strongly relevant, which confirms the spatial dependence directly. The regional heterogeneity tests return negative and significant coefficients in the eastern, central and western regions, so no region contradicts the pooled result, and the northeastern estimate, although not significant, does not differ significantly from that of any other region. And the regional series in Figure 2 move broadly in parallel rather than diverging: from 2013 onward the central region records the lowest compliance share in every year, and attention remains highest in eastern cities and lowest in western cities throughout. Spatially robust standard errors correct inference but do not model spillovers, so a formal spatial Durbin specification with an explicit weight matrix would still be needed to estimate direct and spillover components separately and to establish whether a neighboring city’s air quality affects local attention beyond its effect through local air quality.

The third concerns the dependent measure. The Baidu Index records attention rather than prevalence, and search behavior depends on internet access, age structure and willingness to seek information about mental health. City fixed effects absorb the stable components of these differences and year fixed effects absorb their nationwide evolution, but city-specific changes in search propensity remain a source of measurement error. Linking the index to administrative records of psychiatric service utilization at city rather than provincial level would provide a sharper validation than the correlation reported above.

## Data Availability

The raw data supporting the conclusions of this article will be made available by the authors, without undue reservation.
